# Identification of a carbonic anhydrase–Rubisco complex within the alpha-carboxysome

**DOI:** 10.1073/pnas.2308600120

**Published:** 2023-10-20

**Authors:** Cecilia Blikstad, Eli J. Dugan, Thomas G. Laughlin, Julia B. Turnšek, Mira D. Liu, Sophie R. Shoemaker, Nikoleta Vogiatzi, Jonathan P. Remis, David F. Savage

**Affiliations:** ^a^Department of Molecular and Cell Biology, University of California, Berkeley, CA 94720; ^b^Department of Chemistry - Ångström Laboratory, Uppsala University, Uppsala 75120, Sweden; ^c^Department of Chemistry, University of California, Berkeley, CA 94720; ^d^California Institute for Quantitative Biosciences, University of California, Berkeley, CA 94720; ^e^HHMI, University of California, Berkeley, CA 94720

**Keywords:** CO_2_ fixation, carboxysome, carbonic anhydrase, protein–protein interactions, cryoelectron microscopy

## Abstract

Carboxysomes are proteinaceous organelles that encapsulate key enzymes of CO_2_ fixation—Rubisco and carbonic anhydrase—and are the centerpiece of the bacterial CO_2_ concentrating mechanism (CCM). In the CCM, actively accumulated cytosolic bicarbonate diffuses into the carboxysome and is converted to CO_2_ by carbonic anhydrase, producing a high CO_2_ concentration near Rubisco and ensuring efficient carboxylation. Self-assembly of the α-carboxysome is orchestrated by the intrinsically disordered scaffolding protein, CsoS2, which interacts with both Rubisco and carboxysomal shell proteins, but it is unknown how the carbonic anhydrase, CsoSCA, is incorporated into the α-carboxysome. Here, we present the structural basis of carbonic anhydrase encapsulation into α-carboxysomes from *Halothiobacillus neapolitanus*. We find that CsoSCA interacts directly with Rubisco via an intrinsically disordered N-terminal domain. A 1.98 Å single-particle cryoelectron microscopy structure of Rubisco in complex with this peptide reveals that CsoSCA binding is predominantly mediated by a network of hydrogen bonds. CsoSCA's binding site overlaps with that of CsoS2, but the two proteins utilize substantially different motifs and modes of binding, revealing a plasticity of the Rubisco binding site. Our results advance the understanding of carboxysome biogenesis and highlight the importance of Rubisco, not only as an enzyme but also as a central hub for mediating assembly through protein interactions.

## Significance

Rubisco is responsible for the majority of inorganic carbon assimilation on Earth. To ensure efficient CO_2_ fixation, cyanobacteria and many autotrophic proteobacteria concentrate CO_2_ in the carboxysome, a bacterial organelle encapsulating Rubisco and carbonic anhydrase within a protein shell. It remains unknown exactly how this 250+ megadalton protein complex assembles with high fidelity inside cells. Here, we explore the encapsulation mechanism of the carbonic anhydrase, CsoSCA, and demonstrate that it is incorporated into the α-carboxysome via a carbonic anhydrase–Rubisco complex. Our results update the current model for carboxysome biogenesis and inform strategies for engineering CO_2_ concentration mechanisms into crops and industrially relevant microorganisms for improved growth and yields.

Carbonic anhydrase (CA) catalyzes the rapid interconversion between carbon dioxide (CO_2_) and bicarbonate (HCO_3_^−^). Due to the centrality of this reaction in metabolism, CA is an essential protein in all organisms where it has been tested (1–3). In photosynthesis, CA’s role is often to supply the enzyme Ribulose-1,5-bisphosphate carboxylase-oxygenase (Rubisco)—the carboxylase of the Calvin–Benson–Bassham cycle—with CO_2_ to ensure fast fixation (3). Rubisco has modest turnover numbers and fails to distinguish between CO_2_ and the competing off-target substrate of O_2_ (4–6). To overcome Rubisco’s limitation, plants, algae, and some bacteria have evolved different types of CO_2_-concentrating mechanisms (CCMs) which concentrate CO_2_ near Rubisco (7). This ensures saturation of Rubisco's active sites with CO_2_, competitive inhibition of oxygenation, and an increase in overall carbon assimilation rates. Importantly, to understand the role of a CA in a CCM, it is essential to understand enzyme localization and regulation (3).

The bacterial CCM is present in all cyanobacteria and many proteobacteria. It consists of two main components: (I) energy-coupled inorganic carbon transporters that actively accumulate bicarbonate in the cytosol and (II) a proteinaceous bacterial organelle called the carboxysome, which coencapsulates Rubisco and CA within a capsid-like protein shell (8–11). The accumulated HCO_3_^−^ diffuses into the carboxysome where it is rapidly converted to CO_2_ by CA, while diffusion out of the structure is likely restricted by the shell (12, 13). This produces a locally high CO_2_ concentration within the carboxysome and enables efficient Rubisco carboxylation (14).

**Fig. 1.**
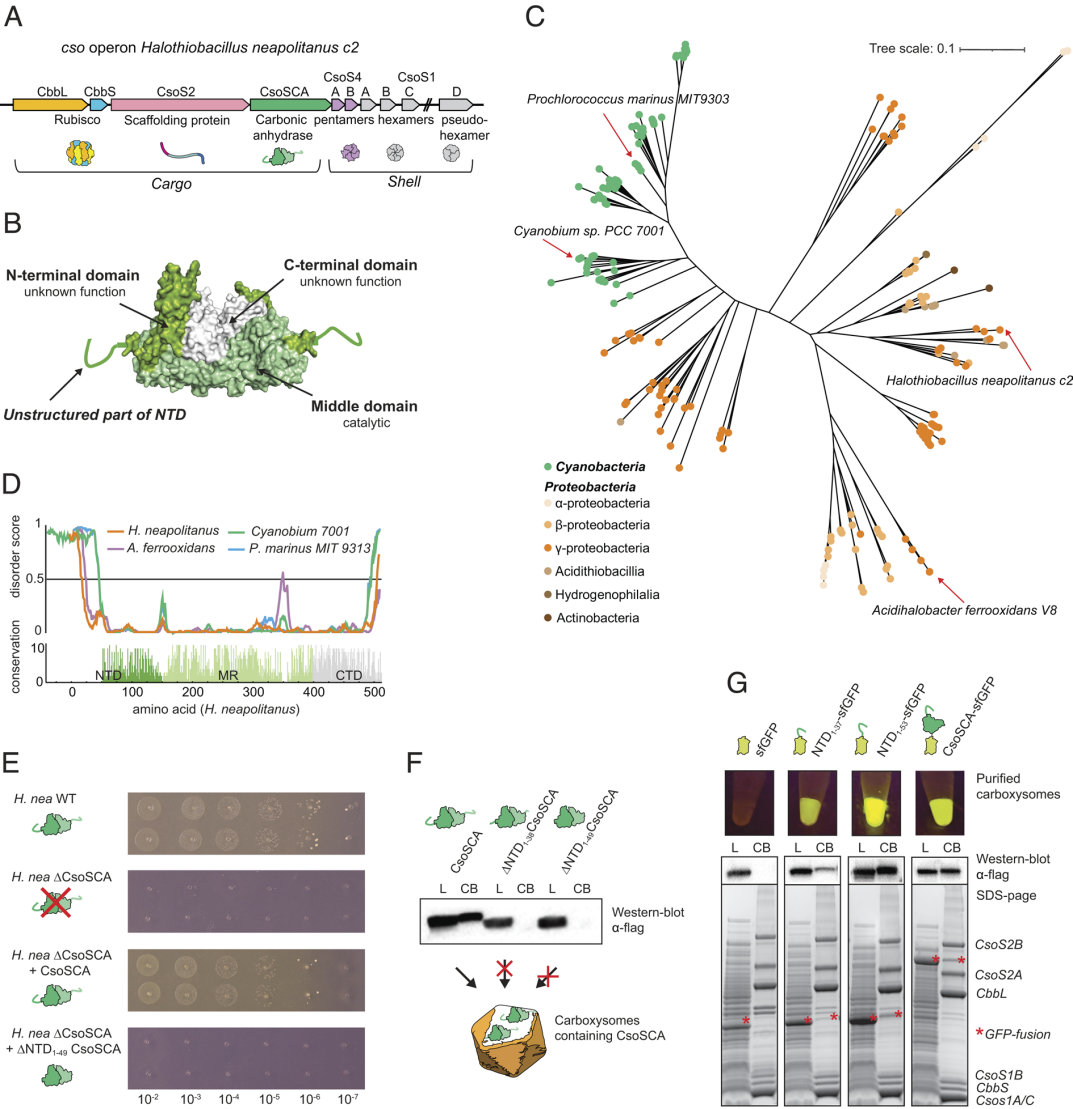
An intrinsically disordered, poorly conserved N-terminal peptide is essential and sufficient for CsoSCA encapsulation. (*A*) Schematic of the cso operon (carboxysome operon) in *H. neapolitanus.* The 10-gene set consists of Rubisco large and small subunits, the scaffolding protein CsoS2, the carbonic anhydrase CsoSCA, and six shell proteins (CsoS4A/B, CsoS1A/B/C, and CsoSD1). In the native organism, CsoS1D is transcribed from an adjacent locus, while in the synthetic pHnCB10 plasmid, all genes are in a single operon. (*B*) Surface representation structure of the CsoSCA dimer from *H. neapolitanus* (pdb: 2FGY). The N-terminal domain (dark green) consists of a ~50-aa-long unstructured peptide followed by a folded α-helical domain with unknown function. The middle domain (light green) contains the active site. The C-terminal domain (white) appears to be a gene duplication of the catalytic domain but lacks essential active site residues. (*C*) Maximum-likelihood phylogenetic tree of CsoSCA. Cyanobacterial homologs are colored in green and proteobacteria homologous in an orange/brown gradient. Scale bar, 0.1 substitutions per site. (*D*) Disorder score of four representative CsoSCA homologous calculated using DISOPRED3 and conservation calculated from multiple sequence alignment. (*E*) Complementation of full-length *csoSCA* rescues growth of a *csoSCA* knock-out in *H. neapolitanus,* while complementation with an NTD-truncated variant, *ΔNTD_1-49_CsoSCA*, fails to rescue growth. (*F*) Western blot analysis detecting C-terminally flag-tagged CsoSCA in lysate (L) and enriched carboxysomes (CB) fractions of carboxysomes produced heterologously in *Escherichia coli*. Synthetic carboxysomes consist of the full *cso* operon (Fig. 1*A*), with either wild-type CsoSCA or an N-terminal truncated variant (ΔNTD_1-37_CsoSCA or ΔNTD_1-49_CsoSCA). CsoSCA is not detected in carboxysomes with N-terminal truncated CsoSCA variants. (*G*) Fusing the unstructured NTD of CsoSCA (37 or 53 residues) to sfGFP targets the fusion protein to synthetic carboxysomes produced heterologously in *E. coli,* while untagged sfGFP does not target to carboxysomes. The control with full-length CsoSCA-sfGFP also produces fluorescent carboxysomes. The panel shows fluorescence of purified carboxysomes, western blot analysis against flag-tagged sfGFP and SDS-PAGE of lysate (L) and purified carboxysomes (CB). (*F* and *G*) The L sample contains detergent for lysing the cells (B-PER II) resulting in a small band shift on the SDS-PAGE, explaining the slightly lower CsoSCA and NTD_1-37_/NTD_1-53_ band in L compared to CB.

Microbiology and biochemistry show there are, in fact, two types of carboxysomes which have evolved convergently (8, 15). These are the α-type found in oceanic cyanobacteria and proteobacteria (Fig. 1*A*) and the β-type found in freshwater cyanobacteria. In both instances, CA localization in the carboxysome is essential for growth in present-day atmospheric CO_2_ concentrations (16–18). In contrast, CA activity in the cytosol has been shown to short-circuit the CCM leading to high CO_2_–requiring phenotypes (19). Efficient encapsulation and regulation of CA activity are thus crucial for cell survival. All α-carboxysomes contain a β-CA, CsoSCA (20–23), while β-carboxysomes have either an active γ-CA domain on the scaffolding protein CcmM (24, 25) or a β-CA named CcaA (26). While the mechanism of CA incorporation into the β-carboxysome is understood (27, 28), it is unknown how CsoSCA incorporates into the α-carboxysome.

CsoSCA belongs to its own subclass of β-CAs and uniquely consists of three domains: an N-terminal domain, a middle/catalytic domain, and a C-terminal domain (Fig. 1*B*) (22, 29). X-ray structural analysis has shown that the catalytic domain contains the zinc-binding site as well as catalytic residues essential for CA activity. The C-terminal domain appears to be an ancient gene duplication of the catalytic domain but lacks the zinc-binding residues. The N-terminal domain (NTD) consists of an unstructured N-terminal peptide followed by a ~100 residue α-helical domain, which lacks homology to any other known protein. The function of this domain is mysterious and has been speculated to be involved in the encapsulation process (22, 29).

Here, we used biolayer interferometry (BLI) to screen CsoSCA for binding to all α-carboxysome proteins and identified Rubisco as its interaction partner. We show that the Rubisco interaction and encapsulation into carboxysomes are dependent on CsoSCA’s unique intrinsically disordered N-terminal peptide. Using this peptide, we targeted foreign cargo into the carboxysome, demonstrating that this sequence is sufficient for encapsulation. We further determined a 1.98 Å single-particle cryoelectron microscopy (cryo-EM) structure of Rubisco in complex with the NTD peptide. The structure reveals that the peptide interacts with Rubisco at a site overlapping with a recently identified site responsible for targeting Rubisco to the α-carboxysome via interaction with the carboxysomal scaffolding protein CsoS2 (30). Thus, our work identifies a carbonic anhydrase–Rubisco supercomplex found inside the α-carboxysome and highlights a surprising flexibility in the scope of protein–protein interactions which lead to α-carboxysome self-assembly.

## Results

### CsoSCA’s N terminus Is Necessary and Sufficient for Encapsulation.

#### *H. neapolitanus* growth assay.

In order to identify putative mechanisms for encapsulation of CsoSCA into the carboxysome, we first started with a bioinformatic examination of the CsoSCA protein. Phylogenetic analysis revealed that CsoSCA from cyanobacteria and proteobacteria cluster into two separate subfamilies (Fig. 1*C*, *SI Appendix*, Fig. S1, and Dataset S1). The cyanobacterial subfamily divides into two clusters. The proteobacteria subfamily is more diverse but has three distinct clusters, including a transition cluster more closely related to the cyanobacterial subfamily. Multiple sequence alignment (MSA) and calculated conservation score reveal a poorly conserved N-terminal region of the NTD, while the rest of the protein is highly conserved (Fig. 1*D*). In our model organism, the γ-proteobacterium *H. neapolitanus,* the sequence conservation of CsoSCA starts with residue H51. Using representatives from the different clusters of the phylogenetic tree, it was revealed that this ~30- to 130-amino-acid-long NTD is predicted to be disordered (Fig. 1*D*). Even though the NTD primary sequence is not conserved, its existence among all species is suggestive of a function.

To investigate the role of CsoSCA’s unstructured NTD-peptide, a *csoSCA* deletion strain of *H. neapolitanus* (*ΔCsoSCA*) was complemented with a version of CsoSCA lacking its first 49 residues (*ΔNTD_1-49_CsoSCA)*. The *ΔNTD_1-49_CsoSCA* strain failed to grow in air (Fig. 1*E*), whereas complementation with full-length CsoSCA rescued growth, indicating an essential role for CsoSCA’s NTD. Synthetic carboxysome operons containing truncated CsoSCAs (*ΔNTD_1-37_CsoSCA* and *ΔNTD_1-49_CsoSCA*) were heterologously expressed in *E. coli*. Western blot analysis of enriched carboxysome (Fig. 1*F*) fractions showed that neither of these constructs produced carboxysomes containing CsoSCA, suggesting that the growth defect seen in the phenotyping experiment is due to an inability to encapsulate CsoSCA into the carboxysome when the N-terminal peptide (NTD_1-49_-peptide) is removed.

To further test the role of the CsoSCA NTD in encapsulation, we sought to target foreign cargo into the carboxysome via fusion with peptides derived from the NTD. Monomeric superfolder GFP (sfGFP) fused with either the first 37 (NTD_1-37_-sfGFP) or first 53 (NTD_1-53_-sfGFP) residues of CsoSCA NTD was coexpressed with synthetic carboxysomes in *E. coli* and purified to assess GFP encapsulation. Both NTD_1-37_-sfGFP and NTD_1-53_-sfGFP produced green fluorescent carboxysomes containing the fusion protein (Fig. 1*G*), while a negative control did not. Normalized for expression levels, the encapsulation efficiencies (sfGFP fluorescence of carboxysome/lysate) were as follows: NTD_1-37_-sfGFP: ~3%, NTD_1-53_-sfGFP: ~15% and CsoSCA-sfGFP: ~23% (Dataset S2). This indicates that additional sequence elements not present in NTD_1-37_-sfGFP may be needed for efficient encapsulation. Shell and Rubisco protein levels were the same for all carboxysome constructs and, hence, should not influence the efficiency of NTD encapsulation (Dataset S2). In summary, these results demonstrate that the N-terminus is necessary and sufficient for encapsulating CsoSCA into the carboxysome.

### CsoSCA Interacts with Rubisco.

**Fig. 2.**
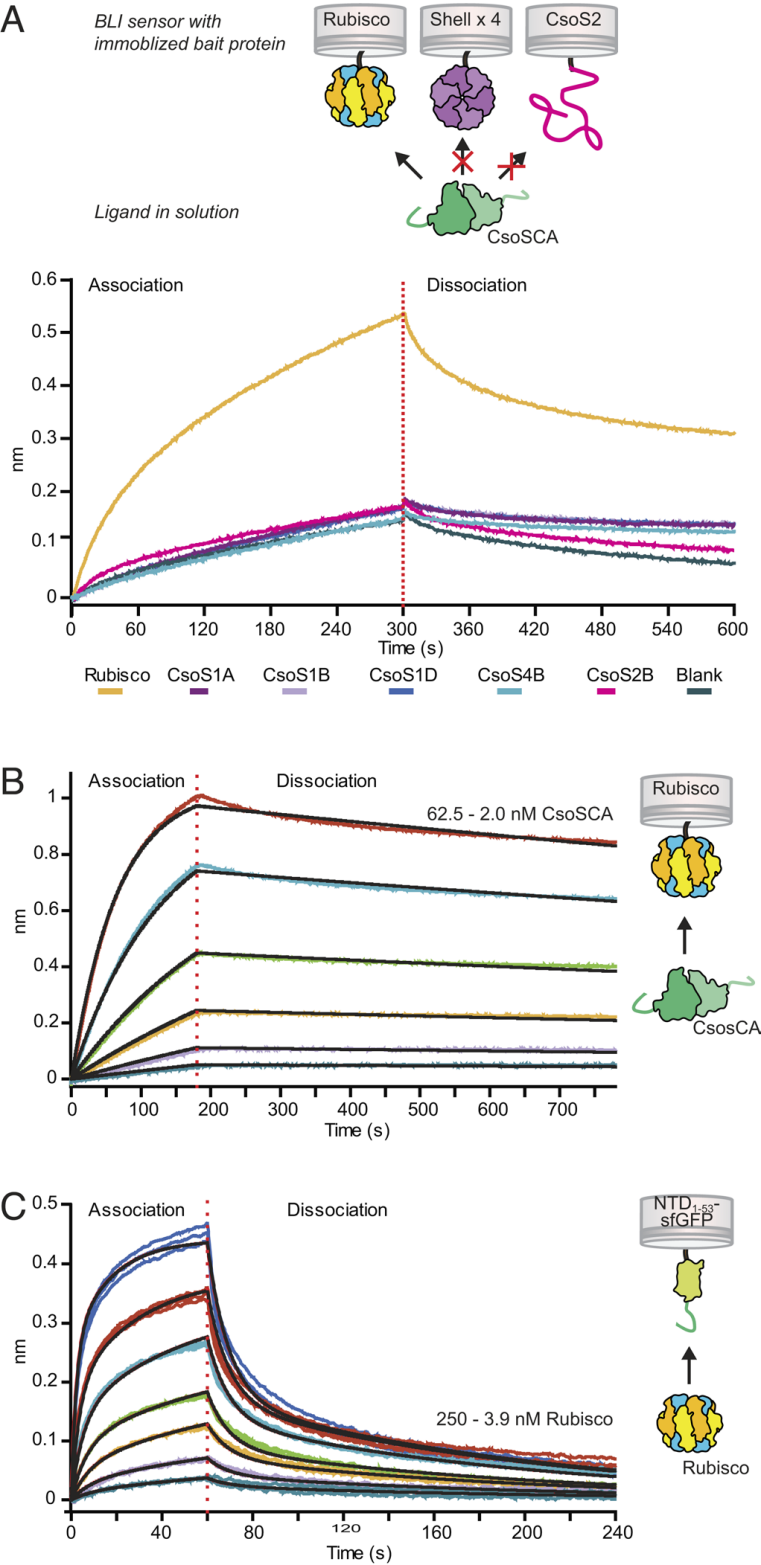
CsoSCA interacts with Rubisco via its N-terminal peptide. (*A*) Biolayer interferometry binding screen with CsoSCA against carboxysome proteins. Binding of CsoSCA (green) was assayed against Rubisco (yellow), the shell proteins; CsoS1A (purple), CsoS1B (light purple), CsoS1D (blue), and CsoS2B (light blue) and against the scaffolding protein CsoS2B (magenta). BLI responses showed binding to Rubisco, while none of the other carboxysome proteins showed detectable binding. (*B*) BLI response from binding affinity measurement of CsoSCA-MBP against immobilized Rubisco. CsoSCA concentration ranged from 62.5 to 2.0 nM in a 1:2 dilution series. (*C*) BLI response from binding affinity measurements of Rubisco against immobilized NTD_1-53_-sfGFP (light green). Rubisco concentration ranged from 250 to 3.9 nM in a 1:2 dilution series.

Previous immunogold labeling EM and biochemical assays (freeze/thaw treatment of carboxysomes) have suggested that CsoSCA may associate with the shell, but no specific interactions have been described (20, 21). Thus, to identify CsoSCA’s interaction partner, we measured binding of untagged CsoSCA against most carboxysome proteins (the shell proteins CsoS1A, CsoS1B, CsoS1D, and CsoS4B; the scaffolding protein CsoS2B; and Rubisco) using BLI. This screen showed that CsoSCA interacted with Rubisco, while none of the other carboxysome proteins had detectable binding above background (Fig. 2*A*). An N-terminal truncated protein variant (ΔNTD_1-37_CsoSCA) did not bind Rubisco, confirming NTD’s involvement in the interaction (*SI Appendix*, Fig. S2*A*). Native-PAGE confirmed binding between CsoSCA and Rubisco and lack of binding to the major shell proteins CsoS1A and CsoS1B (*SI Appendix*, Fig. S2*B*). Finally, coelution of Rubisco and NTD_1-53_-sfGFP using size exclusion chromatography confirmed the interaction in a solution-based assay (*SI Appendix*, Fig. S2*C*).

Concentration dependence of CsoSCA binding to Rubisco was confirmed by BLI and *K*_D_ of the interaction determined to be 1.2 nM ± 0.1 (*k*_on_ = 2.5 × 10^5^ M^−1^s^−1^, *k*_off_ = 2.9 × 10^−4^ s^−1^) (Fig. 2*B* and Table 1). Due to instability of WTCsoSCA, the *K*_D_ was measured with a CsoSCA-MBP fusion [the negative control of MBP alone did not bind Rubisco (*SI Appendix*, Fig. S2*D*)]. Using the stopped-flow based Khalifah/pH-indicator assay (31), CsoSCA-MBP was confirmed to be catalytically active (*SI Appendix*, Fig. S2*G*), demonstrating retained physiological state of the fusion protein. Although a mutated variant of CsoSCA crystallized as a dimer (Fig. 1*B*) (Y92H mutation resulting in introduction of an additional Zn^2+^-binding site) (22), the CsoSCA-MBP protein eluted on size exclusion chromatography at an estimated molecular weight of 612 kDa, suggesting a hexameric state (*SI Appendix*, Fig. S2 *E* and *F*). Due to this discrepancy, present data cannot conclude whether WTCsoSCA is dimeric or hexameric. Rubisco binding to NTD_1-53_-sfGFP further confirmed the NTD_1-53_–peptide interaction (*K*_D1_ = 30 nM, *K*_D2_ = 80 nM; fit to a 1:2 model) (Fig. 2C, Table 1, and *SI Appendix*, Fig. S2*G*). The 25-fold higher *K*_D_ with CsoSCA compared to NTD_1-53_-sfGFP is mainly an effect of a slower off-rate, demonstrating the importance of the multivalency (resulting from CsoSCA being multivalent (dimeric or hexameric) while NTD_1-53_-sfGFP is monomeric) in obtaining a high-affinity interaction with Rubisco.

**Table 1.** Binding constants and kinetics for CsoSCA and NTD_1-53_-sfGFP binding to Rubisco measured with BLI

Immobilized	In solution	*K*_D_ (nM)	*K*_D2_ (nM)	*k*_on_ (M^−1^s^−1^)	*k*_on2_ (M^−1^s^−1^)	*k*_off_ (s^−1^)	*k*_off2_ (s^−1^)
Rubisco	CsoSCA-MBP	1.2 ± 0.1		2.5 × 10^5^ ± 1 × 10^2^		2.9 × 10^−4^ ± 2 × 10^−5^	
NTD_1-53_-sfGFP	Rubisco	33 ± 0.4	80 ± 1	2.1 × 10^5^ ± 2 × 10^3^	1.4 × 10^6^ ± 1 × 10^4^	6.9 × 10^−3^ ± 2 × 10^−5^	1.1 × 10^−1^ ± 5 × 10^−4^
NTD_1-53_-sfGFP R23A	Rubisco	17 ± 0.2	130 ± 2	1.9 × 10^5^ ± 2 × 10^3^	4.6 × 10^5^ ± 8 × 10^3^	3.3 × 10^−3^ ± 2 × 10^−5^	6.1 × 10^−2^ ± 5 × 10^−4^
NTD_1-53_-sfGFP	Rubisco D99A	400 ± 10	1,100 ± 14	1.5 × 10^4^ ± 4 × 10^2^	1.3 × 10^5^ ± 2 × 10^3^	5.9 × 10^−3^ ± 4 × 10^−5^	1.5 × 10^−1^ ± 7 × 10^−4^
NTD_1-53_-sfGFP	Rubisco Y72A	320 ± 6	820 ± 11	2.8 × 10^4^ ± 5 × 10^2^	1.8 × 10^5^ ± 2 × 10^3^	8.9 × 10^−3^ ± 4 × 10^−5^	1.5 × 10^−1^ ± 8 × 10^−4^
NTD_1-53_-sfGFP P22A	Rubisco	18,000 ± 3,000	6,500 ± 400	2.4 × 10^3^ ± 4 × 10^2^	6.8 × 10^4^ ± 4 × 10^3^	4.4 × 10^−2^ ± 3 × 10^−4^	4.4 × 10^−1^ ± 7 × 10^−3^

### Structure of Rubisco in Complex with the CsoSCA NTD Peptide.

An emerging theme of CCM self-assembly is that Rubisco interacts with various CCM proteins via Short Linear Motifs (SLiMs) found in intrinsically disordered proteins/regions (IDP/Rs) (30, 32, 33). Since CsoSCA’s NTD appears to bind Rubisco (Fig. 2*C* and *SI Appendix*, Fig. S3), we next sought to determine the structure of Rubisco in complex with this peptide using cryo-EM. In order to promote high occupancy of available binding sites, an excess of NTD peptide was complexed with Rubisco and imaged as described in the Materials and Methods. These data yielded two slightly distinct single-particle reconstructions of Rubisco bound to a peptide corresponding to the first 50 residues of CsoSCA (NTD_1-50_) at 1.98 Å (State-1) and 2.07 Å (State-2) nominal resolution (*SI Appendix*, Figs. S4 and S5 and Table S1). Of note, both reconstructions show densities for ordered waters and alternate side-chain conformers (*SI Appendix*, Fig. S5). The two confirmations are highly similar (RMSD: 0.22 Å) with the most notable differences occurring in the β-sheet of the N-terminal domain of CbbL, in particular the conformations of loops P37-D42 and G115-G125 (*SI Appendix*, Fig. S6). Since both reconstructions display similar density for the NTD_1-50_ peptide, we do not attribute the difference in confirmation to the presence of the peptide but rather subtle “breathing” of Rubisco. For clarity, we choose to predominantly focus our discussion on the higher resolution 1.98 Å structure (State-1).

**Fig. 3.**
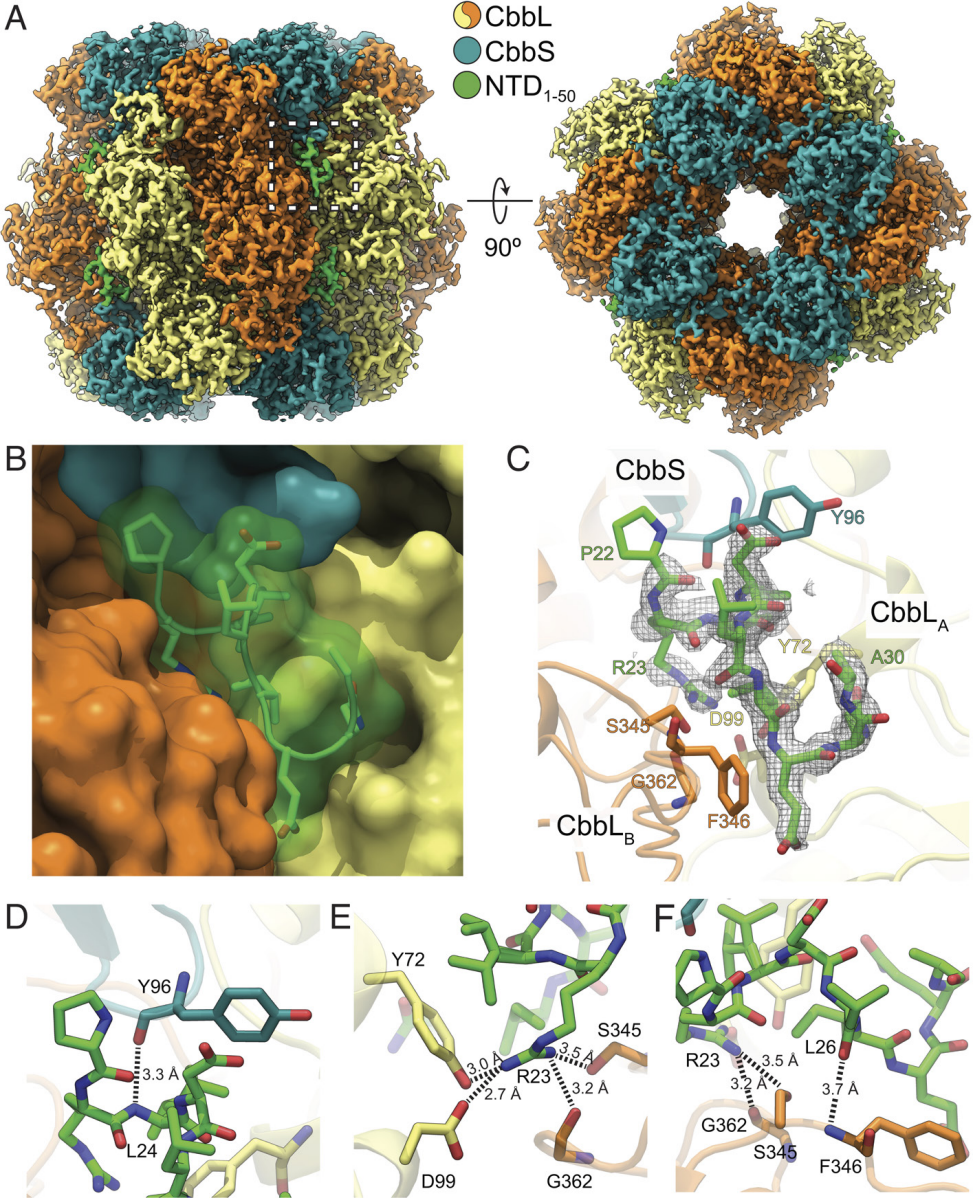
Structure of Rubisco with bound NTD_1-50_ CsoSCA peptide. (*A*) Cryo-EM map of Rubisco bound to a peptide corresponding to the first 50 residues of CsoSCA (NTD_1-50_). The Rubisco–NTD_1-50_ cocomplex is colored by subunit with color key inset. (*B*) Close-up of the region boxed in A of the NTD_1-50_ peptide shown as sticks and transparent surface and Rubisco subunits shown as opaque surfaces. (*C*) Same view as in *B* with NTD_1-50_ peptide and interacting Rubisco residues shown as sticks. NTD_1-50_ peptide density is shown as a gray mesh contoured to 2σ. (*D*–*F*) Detailed polar interactions between residues of NTD_1-50_ peptide and Rubisco are shown as sticks with interaction depicted as dashed lines with distances in Ångströms.

In the cryo-EM reconstruction of the Rubisco–NTD_1-50_ complex, density corresponding to NTD_1-50_ is located in a groove formed at the interfaces of two CbbL subunits (from two different CbbL_2_ dimers) and one CbbS (Fig. 3). The biological assembly of Rubisco is CbbL_8_S_8_, resulting in eight of these binding sites per Rubisco oligomer. The peptide density is of marginally lower-quality (local resolution estimate: 2.1 to 2.2 Å) than that of the surrounding Rubisco density. Nevertheless, we could confidently assign the density to nine residues of the NTD_1-50_ peptide starting at P22 (PRLDLIEQA) (Fig. 3 *B* and *C*). The structure of the Rubisco–NTD_1-50_ complex is highly similar to a previous crystal structure of Rubisco (PDB: 1SVD, RMSD: 0.45 Å, *SI Appendix*, Fig. S7), indicating that binding of the NTD_1-50_ peptide does not induce large-scale conformational changes in Rubisco.

The resolved region of the peptide starts at the bottom of the CbbS subunit (around loop D94-S99), runs downward within the groove between the two CbbL subunits, and ends between β-strand S345-I347 in CbbL_B_ and loop P19-I29 in CbbL_A_, that results in a buried interface of approximately 700 Å^2^. This short stretch of sequence is predicted by JPred to form an alpha-helix (*SI Appendix*, Fig. S8*A*). Indeed, we observe this segment to form a single helical turn and thereafter an extended coil (Fig. 3 *B* and *C*). The sharp turn of the backbone introduced by P22 (the first observed residue of the bound peptide) ensures that the upstream peptide chain points outward toward the solvent instead of clashing with CbbL.

### Binding Is Predominantly Mediated by a Network of Hydrogen Bonds.

Our atomic model of the Rubisco–NTD_1-50_ cocomplex indicates that the interaction between the NTD_1-50_ peptide and Rubisco is largely mediated through polar interactions and, predominantly, hydrogen bonds. R23 forms an extensive network of interactions with the neighboring CbbL subunits. The side chain of R23 forms a salt bridge with D99 (CbbL_A_) and hydrogen bonds to the hydroxyl groups of the CbbL subunits Y72 (CbbL_A_) and S345 (CbbL_B_) as well as to the carbonyl of G362 (CbbL_B_) (Fig. 3*E*). The L24 amide and L26 carbonyl hydrogen bond to the carbonyl of CbbS Y96 and amide of CbbL_B_ F346, respectively (Fig. 3 *D* and *F*).

A water-mediated hydrogen-bonding network likely also contributes to peptide binding (*SI Appendix*, Fig. S9). This putative network is predominantly built up by backbone–water interactions and consists of interactions between N29 and CbbL_A_ Y72 (*SI Appendix*, Fig. S9 *A* and *B*), R23 and CbbL_B_ S345, and L26 and CbbL_B_ F346 (*SI Appendix*, Fig. S9*C*). However, in the lower resolution structure (State-2), the two waters mediating the interactions between the peptide and CbbL_B_ are not resolved (*SI Appendix*, Fig. S9*D*), possibly due to the slightly lower resolution of this reconstruction. Rubisco residues interacting with CsoSCA have a high conservation score among α-carboxysomal Rubiscos but are in general not conserved in β-carboxysomal Rubiscos (*SI Appendix*, Fig. S8*B*).

To determine the relative importance of the different interactions, we measured binding kinetics with a selected set of point mutations on both the NTD peptide and Rubisco (Table 1, Dataset S3, and *SI Appendix*, Fig. S10). The P22A mutation resulted in a dramatic loss in binding. While a protonated CbbS D94 could potentially hydrogen bond with the amide of P22, this large effect is more likely due to P22’s importance in establishing the initial alpha-helical backbone conformation of the peptide or the sharp backbone turn that is essential for binding.

Despite the many interactions made by the buried peptide residue R23, mutation of this residue to alanine yielded roughly the same *K*_D_-value as the wild type. However, mutation of the residues on CbbL_A_ which interact with R23—Y72A and D99A—resulted in a 10-fold increase in *K*_D_ (mainly an effect of slower on-rate). These results are consistent with the net contribution of interactions made by R23 to binding to be quite low, but, nevertheless, this residue adversely affects binding when these interactions are not satisfied in a buried conformation. In this context, R23 may play a role in establishing the specificity of the interaction between Rubisco and CsoSCA.

The remaining hydrogen bonds between the NTD peptide and Rubisco are mediated by backbone moieties. Furthermore, the peptide is tightly packed in the cleft formed by Rubisco to form a buried interface comprising 700 Å^2^ of the 1,200-Å^2^ solvent accessible surface area of the peptide. Thus, given the strong negative effect of the P22A mutant on binding, shape complementarity between peptide and Rubisco appears critical to enable the extensive backbone-mediated hydrogen bonds and van der Waals interactions that drive binding.

### CsoSCA Binds at the Same Site as CsoS2.

**Fig. 4.**
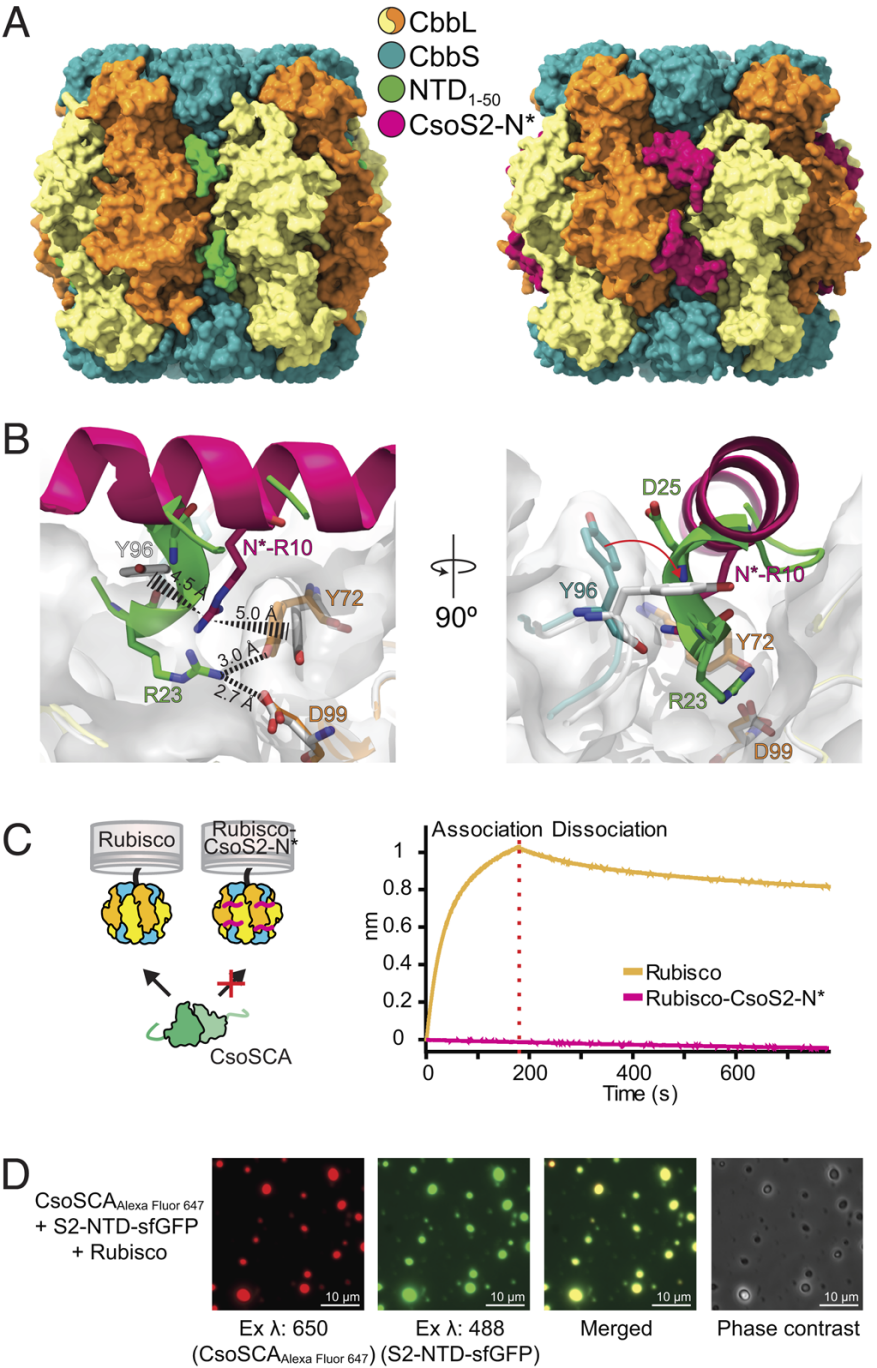
CsoSCA and CsoS2 bind at the same site on Rubisco. (*A*) Surface representation of Rubisco with CsoSCA’s NTD_1-50_ peptide bound and Rubisco with CsoS2 peptide bound (pdb: 6uew). (*B*) Zoomed view of the binding site showing the different conformations of the CsoSCA and CsoS2 peptides. Peptides are shown as cartoons and detailed residues as sticks. Rubisco bound with the NTD_1-50_ peptide is colored according to color key *Inset* in *A* and Rubisco (both subunits) bound with the CsoS2-N* peptide (pdb: 6uew) is colored white. The white transparent surface represents the Rubisco structure which binds CsoSCA. Polar interactions are depicted as dashed lines and cation–pi stacking as dashed triangles with distances in Ångströms. (*C*) The BLI response shows that the CsoS2 peptide fused to Rubisco (Rubisco–CsoS2-N*) passivates binding of CsoSCA to Rubisco. (*D*) Alexa Fluor 647, sfGFP, and merged fluorescence as well as phase contrast images of protein condensates formed from a solution of Rubisco, CsoS2-NTD-sfGFP, and Alexa Fluor 647 labeled CsoSCA-MBP showing that CsoSCA recruits into Rubisco–CsoS2 protein condensates.

α-carboxysome assembly is mediated by a repetitive and disordered protein, CsoS2, which is thought to bind both Rubisco and shell proteins, thus serving as a physical scaffold bridging these two major components. We previously solved the structure of Rubisco in complex with an N-terminal peptide derived from CsoS2 (CsoS2-N*) (30). Surprisingly, CsoSCA and CsoS2 bind at nearly the same location on Rubisco but utilize substantially different SLiMs and binding modes (Fig. 4 *A* and *B*).

CsoS2-N* is largely alpha-helical and binds Rubisco by spanning over the CbbL_2_ dimer interface lying on top of the protein surface (Fig. 4*B*). The complex is highly dependent on salt bridges and cation–pi interactions. In contrast, the CsoSCA peptide is bound in a conformation turned roughly ~45 degrees and with a greater fractional buried surface area for the observed peptide (approximately 700 Å^2^ of 1,200 Å^2^ solvent accessible surface area, compared to 830 Å^2^ of 2,500 Å^2^). CsoSCA is buried deeper into the groove between the two CbbL subunits and interacts mainly via hydrogen bonds and what appears to be an ordered network of water molecules. Notably, both peptides make significant interactions with Rubisco CbbL Y72 (Fig. 4*B*). This residue is conserved in α-carboxysome Rubisco but not in Rubisco from β-carboxysomes or the Form II Rubisco in *H. neapolitanus,* and likely contributes to specificity. Both proteins interact with Y72 via arginines; however, in CsoS2-N*, R10 is cation–pi stacked between CbbL Y72 and CbbS Y96, while in CsoSCA, R23 is positioned deeper into the structure and hydrogen bonds with CbbL Y72 and D99. Another notable feature is CbbS Y96, which in the Rubisco–CsoS2 structure is flipped ~90 degrees compared to the wild-type and Rubisco-CsoSCA structure (Fig. 4*B*), covering the groove between the CbbL subunits interface and enabling the conformation necessary for cation–pi interaction.

Combined, these interactions would seemingly make it impossible for CsoSCA and CsoS2 to bind to the same site of Rubisco at the same time. We have previously developed a Rubisco–CsoS2-N* fusion with all such binding sites occupied due to high local concentration of the CsoS2-N* peptide. As expected, BLI measurement indicated that NTD_1-53_-sfGFP cannot bind to Rubisco when CsoS2-N* is already present, thus confirming that CsoS2 and CsoSCA compete for the same binding site (Fig. 4*C*). Earlier experiments from our group have demonstrated in vitro condensate formation between Rubisco and CsoS2-NTD suggesting that assembly of the α-carboxysome occurs through a condensation-like event (30). Here, we extended these experiments to include CsoSCA in the Rubisco–CsoS2-NTD condensates. These results clearly show that CsoSCA is recruited into the phase-separated Rubisco–CsoS2-NTD condensates (Fig. 4*D*) and demonstrate that all three proteins can simultaneously participate in such a protein interaction network.

## Discussion

In this study, we have determined the structural basis for carbonic anhydrase encapsulation in α-carboxysomes. We found that in the model organism *H. neapolitanus*, CsoSCA and Rubisco form a supercomplex. Through biophysical measurements and in vivo experiments, we found that this complex formation is dependent on the intrinsically disordered N-terminal peptide of CsoSCA. The cryo-EM structure of Rubisco in complex with this peptide reveals that CsoSCA binds Rubisco at a site overlapping with that of the scaffolding protein CsoS2. Aside from its enzymatic activity, this establishes Rubisco’s additional function as an interaction hub in the assembly of the α-carboxysome.

### Updated Model of α-Carboxysome Architecture and Assembly.

**Fig. 5.**
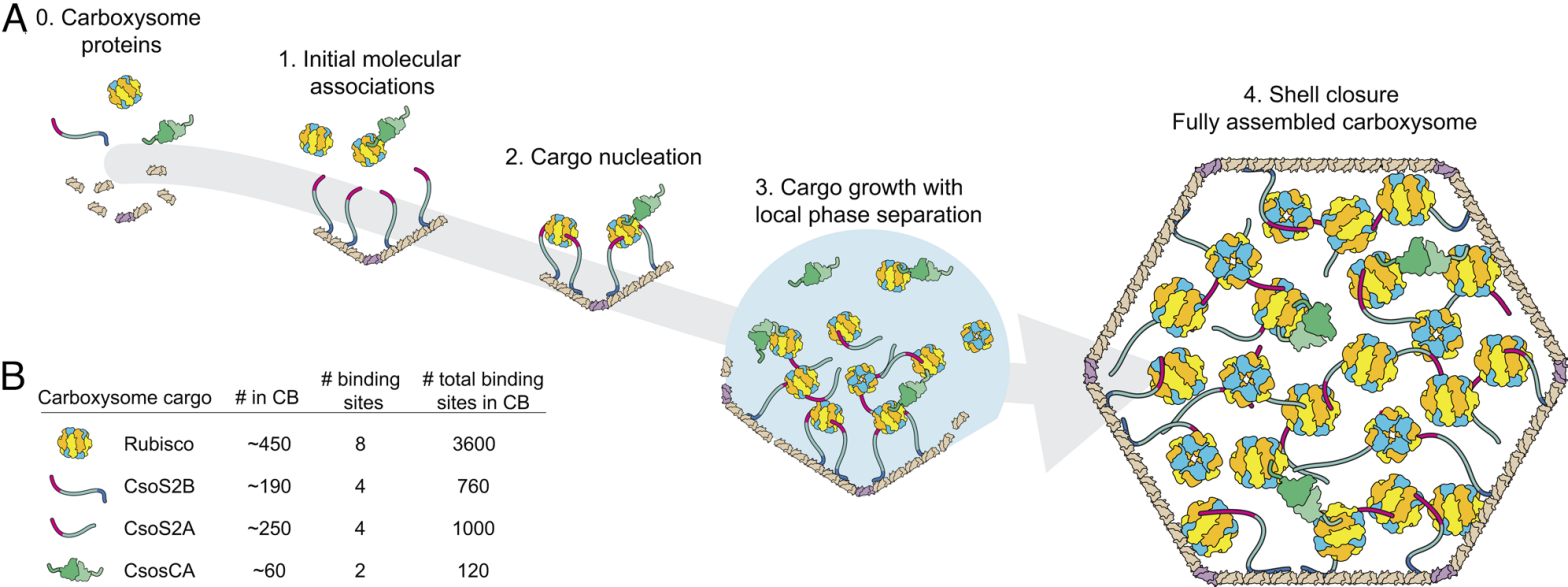
Updated model for carboxysome assembly. (*A*) Schematic model of α-carboxysome assembly in which CsoSCA is recruited to the carboxysome via interactions with Rubisco. The model involves 1) initial molecular associations (specific order not known), 2) Cargo nucleation, 3) cargo growth with local phase separation, and finally 4) shell closure forming fully assembled carboxysomes. Current knowledge does not allow us to distinguish between whether CsoSCA associates with Rubisco during the initial association, step 1, or whether association occurs in the phase-separated condensate, step 3 (or both). CsoSCA is depicted as a dimer; however, present data cannot conclude whether CsoSCA is dimeric or hexameric. The fully assembled carboxysome in step 4 shows a stoichiometrically accurate—with respect to cargo proteins—version of the α-carboxysome. (*B*) Average number of cargo proteins present in an α-carboxysome (36), number of binding sites per oligomeric form of cargo protein, and total number of binding sites per carboxysome.

The intrinsically disordered and repetitive protein CsoS2 acts as a scaffold between shell and Rubisco and orchestrates the assembly (34, 35) of the α-carboxysome. We previously discovered that a repeat of conserved SLiMs (four repeats/protein) found in the N-terminal portion of CsoS2 binds Rubisco and is essential for carboxysome formation (30). Here, we demonstrate that the carboxysomal carbonic anhydrase (CsoSCA) is recruited to the carboxysome via association with Rubisco. We further show that CsoSCA’s N-terminal targeting peptide and CsoS2 bind at the same site on Rubisco. Fig. 5*A* presents our current model of α-carboxysome assembly.

Previous work has shown that, on average, there are 450, 440, and 60 copies of Rubisco, CsoS2, and CsoSCA in a typical *H. neapolitanus* carboxysome, respectively (Fig. 5*B*) (36). The roughly 1,800 CsoS2 and 120 CsoSCA Rubisco binding motifs per carboxysome set an upper boundary of occupancy for the binding sites of Rubisco (~3,600 sites/carboxysome). Assuming all CsoS2 and CsoSCA motifs engage in binding, roughly 50% of Rubisco sites would be occupied by CsoS2, while considerably less, ~3.5%, would be occupied by CsoSCA. Although this assumes that all Rubisco sites are accessible and that all CsoS2 and CsoSCA motifs bind, both assumptions of which could be incorrect, such a calculation indicates there is likely a surplus of Rubisco sites available for binding. Our finding that CsoSCA is recruited to Rubisco–CsoS2-NTD condensates supports the hypothesis that this ternary complex is an important feature of cargo assembly in α-carboxysomes in vivo (Fig. 4*D* and *SI Appendix*, Fig. S11). Further, it has previously been shown that CsoSCA mRNA levels are lower compared to other carboxysome genes (37), suggesting that the amount of encapsulated CsoSCA is likely regulated by protein expression level rather than by competing for binding site occupancy with CsoS2. Due to the need for tight regulation of CA activity outside of the carboxysome, efficient encapsulation is vital (19) and a scenario where CsoSCA had to compete for binding could pose a physiological problem.

One specific unknown is the importance of CsoSCA multivalency imparted by its oligomeric structure and the resulting mode of interaction with Rubisco. The significantly slower dissociation rate of full-length CsoSCA (multivalent) compared to the NTD_1-53_-peptide (monovalent) implies importance of multivalent protein–protein interactions (Table 1 and Fig. 2 *B* and *C*), a feature commonly observed for other IDP/R involved in phase separation (38). In terms of binding mode, bivalent binding of full-length CsoSCA could occur either between two binding sites on the same Rubisco or between two sites on different Rubisco molecules. The relatively short stretch of IDR sequence before the Rubisco binding motif and the rigidity of the folded domains presumably constrains possible binding conformations where CsoSCA binds on top or on the side in a 1:1 CsoSCA–Rubisco complex (*SI Appendix*, Fig. S12). Alternatively, CsoSCA could cross-link two Rubisco molecules (*SI Appendix*, Fig. S12). Further, previous experiments have indicated that CsoSCA is localized to the shell (20, 21). Although our data suggest that CsoSCA makes a primary interaction with Rubisco, and would likely be found throughout the carboxysome, it is possible that additional unknown protein interactions could bias CsoSCA localization toward the shell. Recent cryoelectron tomography work has been unable to unambiguously locate CsoSCA inside the carboxysome (39, 40). However, rapid advances in this technique will likely, in the near future, determine the binding conformation and localization of all such components in situ.

### Plasticity of the Rubisco Binding Motif.

We could not identify a consensus Rubisco SLiM-binding motif across NTD sequences in CsoSCA homologs. The binding element identified in *H. neapolitanus* CsoSCA (PRLDLIEQA) is present in its most closely related homolog (*Halothiobacillus sp. LS2*) but is not conserved across species (further discussed in *SI Appendix*, Fig. S13 *A* and *B*). Many prolines are followed by R or xR, but, overall, the proteobacteria clade contains no convincing conserved motifs. In the cyanobacterial clade, a PTAPx[R/K]R motif is present in 87% of the sequences, suggesting a possible binding motif among cyanobacteria.

Surprisingly, a handful of cyanobacterial CsoSCA sequences from the *Prochlorococcus* genus contain the Rubisco-binding motif found in CsoS2 (RxxxxxRRxxxxxxGK) (*SI Appendix*, Fig. S13*A*), suggesting an evolutionary relationship. The lack of a consensus motif in CsoSCA homologs, coupled with the fact that CsoSCA and CsoS2 bind at the same site but with different mechanisms, reveals an evolutionary plasticity in SLiM sequence space. Across the various microbes in this phylogeny, we hypothesize that CA is recruited to its respective α-carboxysomes by the observed, versatile Rubisco binding site and does so using diverse SLiM sequences.

### Rubisco as an Interaction Hub in Biophysical CCM’s.

Recent work on both bacterial carboxysomes and algal pyrenoids suggests that Rubisco itself plays a role as an interaction hub in the ultrastructural organization of CCMs. It is now clear that not only does Rubisco interact with scaffolding proteins as a means to condensate Rubisco and form these confined CO_2_-fixing organelles, it also recruits auxiliary proteins, such as CAs and activases, needed for the CCM to function. In the bacterial carboxysomal α-lineage, we have demonstrated that Rubisco binds the intrinsically disordered proteins CsoS2 (30) and CsoSCA. Additionally, it also binds the Rubisco activase CbbQO, likely via CbbO’s von Willebrand factor A domain (41, 42). The β-carboxysomal Rubisco binds its interaction partners—the scaffolding protein CcmM (43, 44), and the Rubisco activase Rca (45, 46)—via a folded domain resembling the small subunit of Rubisco (SSLD). Similar to CsoSCA, the β-carboxysomal CA, CcaA, is also recruited via a terminal peptide. However, instead of direct interaction with Rubisco, the two enzymes are linked together via the scaffolding protein CcmM (28). This convergent function may have evolutionary significance—recent results suggest that Rubisco-CA colocalization was an important step in the evolution of biophysical CCMs (47, 48). Despite convergent evolution, a notable similarity between both carboxysome lineages is the binding site on Rubisco. In known cases (except for CbbQO), the interactor binds at the same patch on Rubisco and makes contact with two different CbbL_2_ dimers and one CbbS. This likely ensures that the binding partner only interacts with fully assembled CbbL_8_S_8_ Rubiscos during the assembly process.

In contrast to these bacterial systems, in the model algae *Chlamydomonas reinhardtii*, a repeat SLiM in the disordered scaffolding protein EPYC1 is essential for pyrenoid formation (49, 50) and binds on top of the small subunit via salt bridges and a hydrophobic interface (32). The sequence motif is shared among many pyrenoid proteins, suggesting a mechanism for protein targeting as well as more broadly organizing pyrenoid ultrastructure (33). The versatility in binding motif and binding site, and the convergent function of diverse Rubiscos as a hub of interaction, implies this might be a general feature, which raises a final question: Do Form IB plant Rubiscos engage in similar protein–protein interactions and do other Rubisco Forms also function as interaction hubs?

In summary, this work advances our understanding of carboxysome biogenesis and puts a focus on both the essential carbonic anhydrase and the role of Rubisco as a hub protein. This provides critical findings for engineering the carboxysome-based CCM into, e.g., crops and industrially relevant microorganisms for improved growth and yields. More broadly, we hope that the findings presented here will advance our understanding of bacterial microcompartments and promote development of their many potential biotechnological applications.

## Materials and Methods

### Bioinformatics.

Protein sequences assigned CsoSCA (pfam08936) from all finished and permanent draft bacterial genomes available in the Integrated Microbial Genomes and Microbiomes database (51) were collected on December 12, 2019, and curated to only include proteins in an α-carboxysome operon (containing CbbL/S, CsoS2, and shell hexamers and pentamers) (412 genes). Thereafter, redundancy was reduced by removing sequences with >98% identity using Jalview, and sequences were manually curated to remove incomplete sequences, resulting in 222 sequences in the final CsoSCA dataset (Dataset S1). Sequences were aligned using MUSCLE (52). Resulting MSA was used to calculate the conservation score (53). Tree was built using IQ-TREE web server (54) and visualized using iTOL (55). Protein disorder was predicted for a subset of the dataset, including *H. neapolitanus* CsoSCA, using the DISOPRED3 algorithm (56). Conservation of CsoSCA NTD Rubisco binding motif was analyzed using The MEME Suite (57) (Dataset S1). MSA of α-carboxysomal Form IA Rubiscos (135 cbbL and 132 cbbS sequences) and β-carboxysomal Form IB Rubiscos (211 cbbL and 207 cbbS sequences) was constructed (Dataset S1) using, MUSCLE and visualized with WebLogo. Secondary structure prediction of NTD sequence was performed using Jpred4 (58).

### Protein Expression and Purification.

Specifics regarding *E. coli* strain, plasmid, expression condition, and purification method for each protein in this study (CsoSCA variants, sfGFP fusions, Rubisco, shell proteins and CsoS2) are provided in Dataset S4. Protocols for protein expression and purification are fully described in *SI Appendix*, *Method*. In short, *E. coli* cells harboring appropriate expression plasmids were grown at 37 °C in LB-medium supplemented with appropriate antibiotics. At OD_600_ = 0.4–0.6, the expression was induced, and the cells were grown overnight at 18 °C. All Rubisco constructs were coexpressed with GroEL/ES. All CsoSCA-variants, sfGFP fusions, shell proteins, and CsoS2 were purified by His-tag purification and all Rubisco variants by Strep-tag purification. To obtain pure untagged CsoSCA, purified His-SUMO-CsoSCA was cleaved using Ulp-protease. His-tag purified CsoSCA-MBP was further cleaned up by size exclusion chromatography. See *SI Appendix*, *Methods* for full purification protocols, including purification columns and buffer conditions. Protein purities were assessed by SDS-PAGE and were in general >95% pure. For storage, proteins were made to 10% (w/v) glycerol, flash-frozen in liquid nitrogen, and stored in −80 °C. The oligomeric state of CsoSCA-MBP was determined from the Superose 6 Increase chromatogram and a Gel Filtration Standard (BioRad, #1511901).

### Growth Phenotypes of *H. neapolitanus csoSCA* Mutants.

#### Generation of *H. neapolitanus* *ΔcsoSCA* and of WTcsoSCA and ΔNTD_1-49_csoSCA mutant complementations.

*csoSCA* was knocked out by insertion of a spectinomycin cassette. CsoSCA mutant complementations (*ΔcsoSCA+WTcsoSCA; ΔcsoSCA+NTD_1-49_csoSCA)* were genomically integrated into *H. neapolitanus* NS2 neutral site.

#### H. neapolitanus growth assays.

Precultures of WT *H. neapolitanus* and *H. neapolitanus ΔcsoSCA* were grown in DSMZ68 at 5% CO_2_ supplemented with the appropriate antibiotics. To induce CA expression, 1 μM IPTG was added to *ΔcsoSCA* transformed with wild-type *csoSCA* or the N-terminal truncation *ΔNTD_1-49_csoSCA*. Upon reaching log phase, cultures were spun down, washed twice, and then serially diluted in 10x steps from 10^−1^ to 10^−8^ OD600. Resulting titers were spotted onto plates in 5% CO_2_ and ambient air; strains expressing complemented *WTcsoSCA* or *ΔNTD_1-53_csoSCA* were plated on plates containing 1 μM IPTG. Strains were allowed to grow for 4 d. All strains were plated in biological and technical triplicate. Protocols for *H. neapolitanus* genomic modifications and growth assays are fully described in *SI Appendix*, *Methods*.

### Carboxysome Purifications.

#### Small-scale enrichment of carboxysomes.

First, 100 mL of *E. coli* BW25113 cells harboring pHnCB10 (plasmid for homologous expression of *H. neapolitanus* α-carboxysomes in *E. coli*), containing wild type, ΔNTD_1-37_, or ΔNTD_1-49_ truncation of flag-tagged CsoSCA, were grown at 30 °C in LB medium supplemented with appropriate antibiotics (Dataset S4). At OD_600_ = 0.4–0.6, the expression was then induced by addition of 0.5 mM IPTG, cells grown for 4 h, harvested by centrifugation at 5,000 × *g,* and frozen at −20 °C until use. To enrich carboxysomes, the cell pellets were resuspended and chemically lysed for 30 min in 6 mL of B-PER II (Thermo Fisher) diluted to 1× with TEMB buffer (10 mM Tris pH 8.0, 10 mM MgCl2, 20 mM NaHCO_3_, and 1 mM EDTA) supplemented with 0.1 mg/mL lysozyme, 1 mM PMSF, and 0.1 μL of benzonase/mL (Sigma-Aldrich). Lysed cells were centrifuged 12,000 × *g* for 15 min to remove cell debris. The clarified lysate was centrifuged 40,000 × *g* for 30 min, and the enriched carboxysome fraction were thereafter resuspended in 200 μL of TEMB.

#### Full-scale purification of carboxysomes coexpressed with CsoSCA-sfGFP fusions.

One liter of *E. coli* BW25113 cells cotransformed with pHnCB9 (pHnCB10 lacking the gene for *csoSCA*) and pFA-plasmid containing CsoSCA variants fused to sfGFP (NTD_1-37_-sfGFP, NTD_1-53_-sfGFP, or CsoSCA-sfGFP) were grown at 37 °C in LB-medium supplemented with appropriate antibiotics (Dataset S4). At OD_600_ = 0.4–0.6, the expression was induced by addition of 0.5 mM IPTG and 0.1 μM aTc, temperature decreased to 18 °C, and grown o/n. Thereafter, cells were harvested by centrifugation at 5,000 × *g* and frozen at −20 °C until use. To purify carboxysomes, cells were chemically lysed for 30 min under mild shaking in B-PER II (Thermo Fisher) diluted to 1× with TEMB buffer supplemented with 0.1 mg/mL lysozyme, 1 mM PMSF, and 0.1 μL of benzonase/mL (Sigma-Aldrich). Lysed cells were centrifuged 12,000 × *g* for 15 min to remove cell debris. The clarified lysate was centrifuged 40,000 × *g* for 30 min to pellet carboxysomes. The obtained pellets were gently resuspended in 1.5 mL TEMB buffer, loaded on top of a 25 mL 10 to 50% sucrose step gradient (10, 20, 30, 40, and 50% w/v sucrose, made in TEMB buffer) and ultracentrifuged at 105,000 × *g* for 35 min (SW 32 Ti Swinging-bucket, Beckman Coulter). Gradients were fractionated, analyzed by SDS-PAGE, and carboxysome-containing fractions pooled and ultracentrifuged 100,000 × *g* for 90 min. Resulting pellets were gently resuspended in TEMB to obtain the final purified carboxysome sample.

Final carboxysome samples and the lysate were analyzed for the presence of CsoSCA or sfGFP fusion protein by SDS-PAGE (4 to 20% Mini-PROTEAN® TGX™ Precast Protein Gels, Bio-Rad), western blot, and GFP fluorescence. For western blotting, proteins from SDS-PAGE gels were transferred to nitrocellulose membranes using the Trans-Blot Turbo system (Bio-Rad). Membranes were blocked with 5% (w/v) nonfat dry milk in phosphate-buffered saline (PBS), 0.1% (v/v) Triton X-100 for 1 h at room temperature. Immunolabeling of Flag-tag was done overnight in 4 °C in the above-mentioned buffer containing a 1:5,000 dilution of a monoclonal anti-Flag horseradish peroxidase–conjugated antibody (Sigma). Membranes were washed 3 × 10 min with PBS, 0.1% (v/v) Triton X-100, and blots were thereafter developed using the SuperSignal West Pico Chemiluminescent Substrate (ThermoFisher) according to the manufacturer’s procedure. Gels and western blots were imaged with the ChemiDoc^TM^ XRS+ System (Bio-Rad). Fluorescence of sfGFP samples was quantified using an Infinite M-1000 plate reader (Tecan). To quantify encapsulation efficiency, the ratio of sfGFP fluorescence in carboxysomes (encapsulated protein)/lysate (expressed protein) was used. The ratio of shell/Rubisco content was quantified using densitometry by measuring the intensity of the CsoS1B and CbbS bands on the SDS-PAGE using ImageJ.

### Biolayer Interferometry.

Protein–protein interactions were measured by Biolayer interferometry (BLI) using an Octet RED384 (Forte Bio). Experimental binding sequence used was the following: loading bait 60 to 240 s, buffer wash 60 s, prey association and prey dissociation (followed by sensor regeneration for Ni-NTA sensors).

#### CsoSCA binding screen.

Purified Rubisco, CsoS1A, CsoS1B, CsoS1D, CsoS4B, and CsoS2B were used as bait proteins and screened for CsoSCA binding. Bait proteins were immobilized on Octet® Ni-NTA Biosensors (Forte Bio) via terminal His-tag using: 5 μg/mL Rubisco, 3.5 μg/mL SUMO-S1A, 8 μg/mL SUMO-S1B, 5 μg/mL SUMO-S1D, 1.3 μg/mL SUMO-S4B, or 2.6 μg/mL CsoS2B. To avoid tiling of shell proteins on the sensor surface, SUMO fusions were used for CsoS1A, 1B, 1D, and 4B (59). Also, 1 μM untagged CsoSCA was used as soluble prey protein. A binding assay was performed in a final buffer of 30 mM Tris pH 7.5, 145 mM NaCl, 1.0 mM TCEP, and 0.01% Triton X100. After a run Ni-NTA sensors were regenerated in 50 mM Tris pH 8.0, 300 mM NaCl, 300 mM imidazole, and 0.05% (w/v) SDS and experiment performed in triplicate.

#### NTD_1-53_-sfGFP vs. Rubisco.

Assays were performed as described above using 5 μg/mL NTD_1-53_-sfGFP-His as bait and varied concentration of strep-Rubisco as prey (WT: 250–3.9 nM, for mutants see *SI Appendix*, Fig. S10) in 25 mM Tris pH 7.5, 70 mM NaCl, and 0.01% Triton X100 and performed in triplicate of duplicates. When indicated, point mutants of NTD_1-53_-sfGFP or Rubisco were used.

#### Rubisco vs. CsoSCA.

2.5 mg/mL of biotinylated Rubisco was immobilized as bait protein on Octet® Streptavidin Biosensors (Forte Bio) and 62.5–2.0 nM of C-terminal MBP tagged CsoSCA was used as prey. Experiment was performed in triplicate in 25 mM Tris pH 7.5, 125 mM NaCl, and 0.01% Triton X100.

#### Rubisco vs. CsoSCA’s NTD_1-50_ peptide.

Biotinylated Rubisco was used as bait and the NTD_1-50_ peptide (100, 50, and 10 μM) as prey in a final buffer of 25 mM Tris, pH 7.5, 85 mM NaCl and 0.01% Triton X100.

Binding and kinetic constants were extracted using the Data Analysis HT 10.0.00.44 software in the Octet Forte Bio package. NTD_1-53_-sfGFP vs. Rubisco were fitted to a 1:2 (Bivalent Analyte) binding model and Rubisco vs. CsoSCA to a 1:1 binding model.

### Size Exclusion Chromatography Analysis of the NTD_1-53_-sfGFP:Rubisco Cocomplex.

Purified Rubisco-strep and NTD_1-53_-sfGFP samples were exchanged into moderate-salt buffer (20 mM Tris, pH 7.5, 150 mM NaCl) using Zeba desalting columns. For cocomplexing, the protein samples were mixed at an 32:1 ratio (NTD_1-53_-sfGFP:CbbL_8_S_8_) and incubated briefly on ice prior to injection over a 3.2/300 Superose 6 Increase column equilibrated in 20 mM Tris, pH 7.5, and 150 mM NaCl at 4 °C. The column was eluted isocratically in the same buffer with elution of total protein monitored by A_280_ and elution of sfGFP-containing fractions monitored by A_485_.

### Native-PAGE Analysis of CsoSCA Binding.

Binding of CsoSCA-MBP to Rubisco and shell proteins was analyzed by native-PAGE, using 4 to 15% Mini-PROTEAN® TGX™ Precast Protein Gels (Bio-Rad). Then, 2.5 μM CsoSCA-MBP was mixed with 0.5 μM Rubisco or 5 μM CsoS1A and CsoS1B and incubated for 15 min in RT. Final buffer composition was 50 mM Tris and 150 mM NaCl, pH 7.5.

### Carbonic Anhydrase Kinetics.

CO_2_ hydration catalyzed by CsoSCA-MBP was measured using the Khalifah/pH indicator assay (31) on an Applied Photophysics SX20 stopped-flow spectrophotometer at 25 °C. Saturated CO_2_ solution (34 mM) was prepared by bubbling CO_2_ gas into milli-Q water at 25 °C. To prevent CO_2_ from escaping, a gas-tight Hamilton syringe was used to inject the solution into the stopped-flow drive syringe. MOPS and para-nitrophenol (pNP) were used as buffer–indicator pairs, and change in pH over time was detected at 400 nm using a pathlength of 1 cm. Final experimental conditions after mixing were 50 mM MOPS, pH 7.5 with the ionic strength adjusted to 50 mM with Na_2_SO_4_, 50 µM pNP, 17 mM CO_2_, and 6 µM of CsoSCA-MBP enzyme. Steady-state kinetics was measured in the timeframe of 0.02 to 0.5 s in eight replicates, and progression curves reaching equilibrium were measured for 60 s in triplicates.

### Cryo-EM of the CsoSCA–Rubisco Complex.

First, 0.5 μM Rubisco-strep was mixed with 0.5 mM of NTD_1-50_ peptide (CsoSCA residue 1-50) in 25 mM Tris pH 7.5, 80 mM NaCl containing 2% glycerol, incubated for 20 min at room temperature, and thereafter stored on ice. Then, 3.5 μL of this sample was deposited onto freshly glow-discharged (PELCO easiGlow), Quantifoil R 1.2/1.3 200 mesh Copper TEM grids (Quantifoil Microtools) and blotted for 3 s using a Mark IV Vitrobot (FEI) after a 30-s delay under 100% humidity at 4 °C conditions before freezing in liquid ethane. The complex was visualized in a Talos Arctica (Thermo Fisher Scientific) operating at 200 keV and equipped with a K3 Summit director electron detector (Gatan) in superresolution CDS mode at 57,000×, corresponding to a pixel size of 0.69 Å. In total, 5,742 movies were acquired with the aid of SerialEM* using a defocus range between −0.6 and −1.8 μm and a 3 × 3 multishot image shift pattern. All movies consisted of 50 frames with a total dose of 50 e-/Å^2^. The data collection was monitored using on-the-fly processing in cryoSPARC live (Structura Biotechnology Inc., https://cryosparc.com/live) (60) to monitor microscope performance, micrograph quality, and orientation distribution of the particles on the grid.

### Image Processing.

Superresolution electron micrograph movies were aligned using MOTIONCOR2 (61) from within RELION 3.1 or using the CPU implementation of motion correction within RELION 3.1. CTF estimation was performed using CTFFIND 4.1 (62) from within RELION 3.1. Micrographs were inspected to remove poor-quality images, resulting in the higher-quality selection of 3,932 micrographs. All further processing was done from within RELION 3.1. Laplacian-of-Gaussian autopicking was used on a subset of 200 micrographs to pick approximately 75,000 particles. These particles were extracted from the micrographs with a pixel size of 2.77 Ångström and a box size of 90 pixels. 2D classification was then used to generate a higher-quality subset of particles that were used to generate an initial 3D model by way of Stochastic Gradient Descent. 3D classification of this higher-quality subset of particles gave us a good-quality 3D reference that was then used as a 3D template.

Approximately 1,300,000 particles were picked from the 3932 micrographs using our 3D reference before being extracted with a pixel size of 2.77 and a box size of 90 pixels. The particles were subjected to 3D classification applying D4 symmetry with a soft circular mask, and the best-looking classes comprising 358,785 particles were selected. The particles were then re-extracted with a pixel size of 1.37 Ångström and a box size of 180 pixels before undergoing another round of 3D classification. Again, the best classes comprising 290,762 particles were selected. The particles were re-extracted with a pixel size of 0.91 Ångström and a box size of 312 pixels before being subjected to 3D autorefinement. The refined particles were then 3D classified without additional image alignment, and the best classes comprising 262,882 particles were selected. These particles underwent CTF refinement and Bayesian polishing before being extracted with a larger 410-pixel size box. A few more rounds of 3D classification and 3D refinement, while selecting only the best classes left us with a homogeneous set of 79,562 particles. 3D refinement of this particle set gives a final resolution of 1.98 Ångstöm at Fourier shell correlation (FSC) = 0.143. RELION 3.1 reports a b-factor of about −36 Å^2^ when sharpened with a soft mask.

### Coordinate Model Building and Refinement.

The coordinate models for the two *H. neapolitanus* Rubisco–NTD_1-50_ complex maps were built and refined similarly using a combination of COOT-v0.9.1 (63) and PHENIX-v1.19.1-4122 (64). Maps for this process were obtained by combination of the respective half-maps without filtering. Maps were molecular weight-based density modified and sharpened with *phenix.resolve_cryo_em* and *phenix.auto_sharpen* (65, 66), respectively. For ease of handling, the maps were reboxed to 160 vx^3^ (about 145 Å^3^) for further use. Chains A (CbbL) and D (CbbS) from the *H. neapolitanus* Rubisco–CsoS2 N*-peptide cocrystal structure (PDB ID: 6UEW) (30), stripped of all ligands, were used as initial models for both maps. The initial models were rigid-body docked and manually reworked to fit the maps in COOT and the resolved portion of CsoSCA NTD_1-50_ peptide built de novo. An initial round of *phenix.real_space_refinement* (67) was performed on models consisting of all asymmetric units with NCS constraints enforced, as well as default target bond length and angle restraints, but without secondary structure, rotamer, or Ramachandran restraints. Putative ordered water molecules were then placed interactively in COOT using maps thresholded at 2σ based on the presence of at least 2 hydrogen-bonding partners and the occurrence of the density in both half-maps. Additional rounds of *phenix.real_space_refinement* and manual adjustment in COOT were performed as described above to yield the final coordinate models. For the higher resolution map (State-1), residues V3-E457 of CbbL were modeled with residues V324-E329 truncated to the C_β_ atoms due to poor side-chain density in this region. Similarly, for the lower resolution map (State-2), residues V3-E457 of CbbL were modeled with residues H291-H300 and V323-D331 truncated to the C_β_ atoms. The CbbS and NTD_1-50_ peptide density for both maps were modeled with residues M4-N110 and P22-A30, respectively.

### Visualization and Structural Analysis.

Structural figures were prepared using a combination of PyMOL-v2.5 (Schrödinger, LLC.) and ChimeraX-v1.2.1 (68). Interface analysis to identify interacting residues and to calculate buried surface area was performed using the ePISA-v1.52 web server (69).

### Condensate Formation Assays.

#### Labeling of CsoSCA-MBP.

His-purified CsoSCA-MBP-his was run on a Superose 6 Increase 10/300 GL size exclusion column (GE Healthcare) in 50 mM HEPES, 300 mM NaCl, pH 8 to remove MBP-his contamination. Pooled fractions containing CsoSCA-MBP were labeled with Alexa Fluor 647 NHS Ester dye at a 1:1 ratio of protein to dye for 2 h in the dark at 4 °C. Excess dye was removed via buffer exchange into 50 mM HEPES, 300 mM NaCl, pH 8 on an EconoPac column (BioRad) and concentrated on a 30K cutoff spin column (Thermo Pierce) at 3,500 × *g* for 20 min. Glycerol was added to a final concentration of 10% before flash-freezing the protein.

#### Condensate formation.

All condensate formation experiments were carried out in a final buffer concentration of 50 mM Tris, 20 mM NaCl, pH 7.5. final protein concentrations were as follows: 1 μM Rubisco, 1 μM CsoS2-NTD-sfGFP, and 0.5 μM CsoSCA-MBP. Then, 20 μL of each mixture was loaded onto a gasket fixed to a cover slip (CoverWell Perfusion Chamber 8x9 mm Dia × 0.9 mm Depth, Grace Bio-Labs) and imaged at 100× on a Zeiss Axio Observer Z1 inverted phase contrast microscope. Green channel excitation was 488 nm and emission was 509 nm. Red channel excitation was 650 nm and emission was 673 nm.

## Supplementary Material

Appendix 01 (PDF)

Dataset S01 (XLSX)

Dataset S02 (XLSX)

Dataset S03 (XLSX)

Dataset S04 (XLSX)

## Data Availability

Cryo-EM maps (sharpened, full, and unfiltered halves) and masks have been deposited with the Electron Microscopy Data Bank, and the corresponding atomic coordinate models deposited with the Protein Data Bank for Rubisco-N50 peptide State-1 (EMD-25201 (70), PDB-7SMK (71)) and State-2 (EMD-25228 (72), PDB-7SNV (73)). Plasmids for all protein constructs used (Dataset S4) are deposited and available from Addgene. All protein sequences used for bioinformatics are available in FASTA format in Dataset S1.

## References

[1] C. T. Supuran, Carbonic anhydrases: Novel therapeutic applications for inhibitors and activators. Nat. Rev. Drug Discov. **7**, 168–181 (2008). 18167490 10.1038/nrd2467

[2] C. Merlin, M. Masters, S. McAteer, A. Coulson, Why is carbonic anhydrase essential to Escherichia coli? J. Bacteriol. **185**, 6415–6424 (2003). 14563877 10.1128/JB.185.21.6415-6424.2003 PMC219403

[3] M. R. Badger, G. D. Price, The role of carbonic anhydrase in photosynthesis. Annu. Rev. Plant Physiol. Plant Mol. Biol. **45**, 369–392 (1994).

[4] I. Andersson, Catalysis and regulation in Rubisco. J. Exp. Bot. **59**, 1555–1568 (2008). 18417482 10.1093/jxb/ern091

[5] C. Bathellier , Ribulose 1,5-bisphosphate carboxylase/oxygenase activates O2 by electron transfer. Proc. Natl. Acad. Sci. U.S.A. **117**, 24234–24242 (2020). 32934141 10.1073/pnas.2008824117 PMC7533879

[6] A. I. Flamholz , Revisiting Trade-offs between Rubisco Kinetic Parameters. Biochemistry **58**, 3365–3376 (2019). 31259528 10.1021/acs.biochem.9b00237 PMC6686151

[7] A. Flamholz, P. M. Shih, Cell biology of photosynthesis over geologic time. Curr. Biol. **30**, R490–R494 (2020). 32428488 10.1016/j.cub.2020.01.076

[8] C. A. Kerfeld, M. R. Melnicki, Assembly, function and evolution of cyanobacterial carboxysomes. Curr. Opin. Plant Biol. **31**, 66–75 (2016). 27060669 10.1016/j.pbi.2016.03.009

[9] B. D. Rae, B. M. Long, M. R. Badger, G. D. Price, Functions, compositions, and evolution of the two types of carboxysomes: Polyhedral microcompartments that facilitate CO2 fixation in cyanobacteria and some proteobacteria. Microbiol. Mol. Biol. Rev. **77**, 357–379 (2013). 24006469 10.1128/MMBR.00061-12 PMC3811607

[10] J. J. Desmarais , DABs are inorganic carbon pumps found throughout prokaryotic phyla. Nat. Microbiol. **4**, 2204–2215 (2019). 31406332 10.1038/s41564-019-0520-8 PMC10184468

[11] A. I. Flamholz , Functional reconstitution of a bacterial CO2 concentrating mechanism in Escherichia coli. ELife **9**, e59882(2020). 33084575 10.7554/eLife.59882 PMC7714395

[12] F. Cai , The pentameric vertex proteins are necessary for the icosahedral carboxysome shell to function as a CO2 leakage barrier. PLoS One **4**, e7521 (2009). 19844578 10.1371/journal.pone.0007521 PMC2760150

[13] N. Mangan, M. Brenner, Systems analysis of the CO2 concentrating mechanism in cyanobacteria. ELife **3**, e02043 (2014). 24842993 10.7554/eLife.02043 PMC4027813

[14] N. M. Mangan, A. Flamholz, R. D. Hood, R. Milo, D. F. Savage, pH determines the energetic efficiency of the cyanobacterial CO2 concentrating mechanism. Proc. Natl. Acad. Sci. U.S.A. **113**, E5354–E5362 (2016). 27551079 10.1073/pnas.1525145113 PMC5018799

[15] M. R. Melnicki, M. Sutter, C. A. Kerfeld, Evolutionary relationships among shell proteins of carboxysomes and metabolosomes. Curr. Opin. Microbiol. **63**, 1–9 (2021). 34098411 10.1016/j.mib.2021.05.011 PMC8525121

[16] M. S. Kimber, Carboxysomal carbonic anhydrases. Subcell Biochem. **75**, 89–103 (2014). 24146376 10.1007/978-94-007-7359-2_6

[17] Z. Dou , CO2 fixation kinetics of Halothiobacillus neapolitanus mutant carboxysomes lacking carbonic anhydrase suggest the shell acts as a diffusional barrier for CO2. J. Biol. Chem. **283**, 10377–10384 (2008). 18258595 10.1074/jbc.M709285200

[18] H. Fukuzawa, E. Suzuki, Y. Komukai, S. Miyachi, A gene homologous to chloroplast carbonic anhydrase (icfA) is essential to photosynthetic carbon dioxide fixation by Synechococcus PCC7942. Proc. Natl. Acad. Sci. U.S.A. **89**, 4437–4441 (1992). 1584776 10.1073/pnas.89.10.4437 PMC49097

[19] G. D. Price, M. R. Badger, Expression of human carbonic anhydrase in the cyanobacterium synechococcus PCC7942 creates a high CO(2)-requiring phenotype: Evidence for a central role for carboxysomes in the CO(2) concentrating mechanism. Plant Physiol. **91**, 505–513 (1989). 16667062 10.1104/pp.91.2.505 PMC1062030

[20] S. H. Baker, D. S. Williams, H. C. Aldrich, A. C. Gambrell, J. M. Shively, Identification and localization of the carboxysome peptide Csos3 and its corresponding gene in Thiobacillus neapolitanus. Arch. Microbiol. **173**, 278–283 (2000). 10816046 10.1007/s002030000141

[21] A.K.-C. So , A novel evolutionary lineage of carbonic anhydrase (epsilon class) is a component of the carboxysome shell. J. Bacteriol. **186**, 623–630 (2004). 14729686 10.1128/JB.186.3.623-630.2004 PMC321498

[22] M. R. Sawaya , The structure of beta-carbonic anhydrase from the carboxysomal shell reveals a distinct subclass with one active site for the price of two. J. Biol. Chem. **281**, 7546–7555 (2006). 16407248 10.1074/jbc.M510464200

[23] S. Heinhorst , Characterization of the carboxysomal carbonic anhydrase CsoSCA from Halothiobacillus neapolitanus. J. Bacteriol. **188**, 8087–8094 (2006). 17012396 10.1128/JB.00990-06 PMC1698195

[24] K. L. Peña, S. E. Castel, C. de Araujo, G. S. Espie, M. S. Kimber, Structural basis of the oxidative activation of the carboxysomal gamma-carbonic anhydrase, CcmM. Proc. Natl. Acad. Sci. U.S.A. **107**, 2455–2460 (2010). 20133749 10.1073/pnas.0910866107 PMC2823891

[25] C. de Araujo , Identification and characterization of a carboxysomal γ-carbonic anhydrase from the cyanobacterium Nostoc sp. PCC 7120. Photosyn. Res. **121**, 135–150 (2014). 10.1007/s11120-014-0018-4 24907906

[26] L. D. McGurn , The structure, kinetics and interactions of the β-carboxysomal β-carbonic anhydrase, CcaA. Biochem. J. **473**, 4559–4572 (2016). 27729545 10.1042/BCJ20160773

[27] B. M. Long, M. R. Badger, S. M. Whitney, G. D. Price, Analysis of carboxysomes from Synechococcus PCC7942 reveals multiple Rubisco complexes with carboxysomal proteins CcmM and CcaA. J. Biol. Chem. **282**, 29323–29335 (2007). 17675289 10.1074/jbc.M703896200

[28] K. Zang, H. Wang, F. U. Hartl, M. Hayer-Hartl, Scaffolding protein CcmM directs multiprotein phase separation in β-carboxysome biogenesis. Nat. Struct. Mol. Biol. **28**, 909–922 (2021). 34759380 10.1038/s41594-021-00676-5 PMC8580825

[29] S. B. Pulsford , Cyanobacterial α-carboxysome carbonic anhydrase is allosterically regulated by the Rubisco substrate RuBP. BioRxiv [Preprint] (2023), 10.1101/2023.07.31.551272 (Accessed 1 August 2023). PMC11086599 38728392

[30] L. M. Oltrogge , Multivalent interactions between CsoS2 and Rubisco mediate α-carboxysome formation. Nat. Struct. Mol. Biol. **27**, 281–287 (2020). 32123388 10.1038/s41594-020-0387-7 PMC7337323

[31] R. G. Khalifah, The carbon dioxide hydration activity of carbonic anhydrase. I. Stop-flow kinetic studies on the native human isoenzymes B and C. J. Biol. Chem. **246**, 2561–2573 (1971). 4994926

[32] S. He , The structural basis of Rubisco phase separation in the pyrenoid. Nat. Plants **6**, 1480–1490 (2020). 33230314 10.1038/s41477-020-00811-y PMC7736253

[33] M. T. Meyer , Assembly of the algal CO2-fixing organelle, the pyrenoid, is guided by a Rubisco-binding motif. Sci. Adv. **6**, eabd2408 (2020). 33177094 10.1126/sciadv.abd2408 PMC7673724

[34] F. Cai , Advances in understanding carboxysome assembly in prochlorococcus and synechococcus implicate csos2 as a critical component. Life (Basel) **5**, 1141–1171 (2015). 25826651 10.3390/life5021141 PMC4499774

[35] T. Chaijarasphong , Programmed ribosomal frameshifting mediates expression of the α-carboxysome. J. Mol. Biol. **428**, 153–164 (2016). 26608811 10.1016/j.jmb.2015.11.017

[36] Y. Sun , Decoding the absolute stoichiometric composition and structural plasticity of α-carboxysomes. MBio **13**, e0362921 (2022). 35343789 10.1128/mbio.03629-21 PMC9040747

[37] F. Cai, S. Heinhorst, J. M. Shively, G. C. Cannon, Transcript analysis of the Halothiobacillus neapolitanus cso operon. Arch. Microbiol. **189**, 141–150 (2008). 17899012 10.1007/s00203-007-0305-y

[38] V. N. Uversky, Intrinsically disordered proteins in overcrowded milieu: Membrane-less organelles, phase separation, and intrinsic disorder. Curr. Opin. Struct. Biol. **44**, 18–30 (2017). 27838525 10.1016/j.sbi.2016.10.015

[39] L. A. Metskas , Rubisco forms a lattice inside alpha-carboxysomes. Nat. Commun. **13**, 4863 (2022). 35982043 10.1038/s41467-022-32584-7 PMC9388693

[40] T. Ni , Structure and assembly of cargo Rubisco in two native α-carboxysomes. Nat. Commun. **13**, 4299 (2022). 35879301 10.1038/s41467-022-32004-w PMC9314367

[41] Y.-C.C. Tsai, M. C. Lapina, S. Bhushan, O. Mueller-Cajar, Identification and characterization of multiple rubisco activases in chemoautotrophic bacteria. Nat. Commun. **6**, 8883 (2015). 26567524 10.1038/ncomms9883 PMC4660213

[42] M. Sutter , Structural characterization of a newly identified component of α-carboxysomes: The AAA+ domain protein CsoCbbQ. Sci. Rep. **5**, 16243 (2015). 26538283 10.1038/srep16243 PMC4633670

[43] H. Wang , Rubisco condensate formation by CcmM in β-carboxysome biogenesis. Nature **566**, 131–135 (2019). 30675061 10.1038/s41586-019-0880-5

[44] P. Ryan , The small RbcS-like domains of the β-carboxysome structural protein CcmM bind RubisCO at a site distinct from that binding the RbcS subunit. J. Biol. Chem. **294**, 2593–2603 (2019). 30591587 10.1074/jbc.RA118.006330 PMC6393606

[45] S. Lechno-Yossef , Cyanobacterial carboxysomes contain an unique rubisco-activase-like protein. New Phytol. **225**, 793–806 (2020). 31518434 10.1111/nph.16195

[46] M. Flecken , Dual functions of a rubisco activase in metabolic repair and recruitment to carboxysomes. Cell **183**, 457–473.e20 (2020). 32979320 10.1016/j.cell.2020.09.010

[47] B. M. Long, B. Förster, S. B. Pulsford, G. D. Price, M. R. Badger, Rubisco proton production can drive the elevation of CO2 within condensates and carboxysomes. Proc. Natl. Acad. Sci. U.S.A. **118**, e2014406118 (2021). 33931502 10.1073/pnas.2014406118 PMC8106323

[48] A. I. Flamholz , Trajectories for the evolution of bacterial CO2-concentrating mechanisms. Proc. Natl. Acad. Sci. U.S.A. **119**, e2210539119 (2022). 36454757 10.1073/pnas.2210539119 PMC9894237

[49] L. C. M. Mackinder , A repeat protein links Rubisco to form the eukaryotic carbon-concentrating organelle. Proc. Natl. Acad. Sci. U.S.A. **113**, 5958–5963 (2016). 27166422 10.1073/pnas.1522866113 PMC4889370

[50] T. Wunder, S. L. H. Cheng, S.-K. Lai, H.-Y. Li, O. Mueller-Cajar, The phase separation underlying the pyrenoid-based microalgal Rubisco supercharger. Nat. Commun. **9**, 5076 (2018). 30498228 10.1038/s41467-018-07624-w PMC6265248

[51] V. M. Markowitz , IMG: The Integrated Microbial Genomes database and comparative analysis system. Nucleic Acids Res. **40**, D115–D122 (2012). 22194640 10.1093/nar/gkr1044 PMC3245086

[52] F. Madeira , The EMBL-EBI search and sequence analysis tools APIs in 2019. Nucleic Acids Res. **47**, W636–W641 (2019). 30976793 10.1093/nar/gkz268 PMC6602479

[53] A. M. Waterhouse, J. B. Procter, D. M. A. Martin, M. Clamp, G. J. Barton, Jalview Version 2–A multiple sequence alignment editor and analysis workbench. Bioinformatics **25**, 1189–1191 (2009). 19151095 10.1093/bioinformatics/btp033 PMC2672624

[54] J. Trifinopoulos, L.-T. Nguyen, A. von Haeseler, B. Q. Minh, W-IQ-TREE: A fast online phylogenetic tool for maximum likelihood analysis. Nucleic Acids Res. **44**, W232–W235 (2016). 27084950 10.1093/nar/gkw256 PMC4987875

[55] I. Letunic, P. Bork, Interactive Tree Of Life (iTOL) v5: An online tool for phylogenetic tree display and annotation. Nucleic Acids Res. **49**, W293–W296 (2021). 33885785 10.1093/nar/gkab301 PMC8265157

[56] D. T. Jones, D. Cozzetto, DISOPRED3: Precise disordered region predictions with annotated protein-binding activity. Bioinformatics **31**, 857–863 (2015). 25391399 10.1093/bioinformatics/btu744 PMC4380029

[57] T. L. Bailey, J. Johnson, C. E. Grant, W. S. Noble, The MEME suite. Nucleic Acids Res. **43**, W39–W49 (2015). 25953851 10.1093/nar/gkv416 PMC4489269

[58] A. Drozdetskiy, C. Cole, J. Procter, G. J. Barton, JPred4: A protein secondary structure prediction server. Nucleic Acids Res. **43**, W389–W394 (2015). 25883141 10.1093/nar/gkv332 PMC4489285

[59] A. R. Hagen , In vitro assembly of diverse bacterial microcompartment shell architectures. Nano Lett. **18**, 7030–7037 (2018). 30346795 10.1021/acs.nanolett.8b02991 PMC6309364

[60] A. Punjani, J. L. Rubinstein, D. J. Fleet, M. A. Brubaker, cryoSPARC: Algorithms for rapid unsupervised cryo-EM structure determination. Nat. Methods **14**, 290–296 (2017). 28165473 10.1038/nmeth.4169

[61] J. Zivanov , New tools for automated high-resolution cryo-EM structure determination in RELION-3. ELife **7**, e42166 (2018). 30412051 10.7554/eLife.42166 PMC6250425

[62] A. Rohou, N. Grigorieff, CTFFIND4: Fast and accurate defocus estimation from electron micrographs. J. Struct. Biol. **192**, 216–221 (2015). 26278980 10.1016/j.jsb.2015.08.008 PMC6760662

[63] A. Casañal, B. Lohkamp, P. Emsley, Current developments in Coot for macromolecular model building of Electron Cryo-microscopy and Crystallographic Data. Protein Sci. **29**, 1069–1078 (2020). 31730249 10.1002/pro.3791 PMC7096722

[64] D. Liebschner , Macromolecular structure determination using X-rays, neutrons and electrons: Recent developments in Phenix. Acta Crystallogr. D Struct. Biol. **75**, 861–877 (2019). 31588918 10.1107/S2059798319011471 PMC6778852

[65] T. C. Terwilliger, S. J. Ludtke, R. J. Read, P. D. Adams, P. V. Afonine, Improvement of cryo-EM maps by density modification. Nat. Methods **17**, 923–927 (2020). 32807957 10.1038/s41592-020-0914-9 PMC7484085

[66] T. C. Terwilliger, O. V. Sobolev, P. V. Afonine, P. D. Adams, Automated map sharpening by maximization of detail and connectivity. Acta Crystallogr. D Struct. Biol. **74**, 545–559 (2018). 29872005 10.1107/S2059798318004655 PMC6096490

[67] P. V. Afonine , Real-space refinement in PHENIX for cryo-EM and crystallography. Acta Crystallogr. D Struct. Biol. **74**, 531–544 (2018). 29872004 10.1107/S2059798318006551 PMC6096492

[68] T. D. Goddard , UCSF ChimeraX: Meeting modern challenges in visualization and analysis. Protein Sci. **27**, 14–25 (2018). 28710774 10.1002/pro.3235 PMC5734306

[69] E. Krissinel, K. Henrick, Inference of macromolecular assemblies from crystalline state. J. Mol. Biol. **372**, 774–797 (2007). 17681537 10.1016/j.jmb.2007.05.022

[70] C. Blikstad , EMD-25201. Electron Microscopy Data Bank. https://www.ebi.ac.uk/emdb/EMD-25201. Deposited 26 October 2021.

[71] C. Blikstad , 7SMK, H. neapolitanus carboxysomal rubisco/CsoSCA-peptide (1-50)complex. Protein Data Bank. https://www.rcsb.org/structure/7SMK. Deposited 26 October 2021.

[72] C. Blikstad , EMD-25228. H. neapolitanus carboxysomal rubisco/CsoSCA-peptide (1-50)complex. Electron Microscopy Data Bank. https://www.ebi.ac.uk/emdb/EMD-25228. Deposited 26 October 2021.

[73] C. Blikstad , 7SNV, H. neapolitanus carboxysomal rubisco/CsoSCA-peptide (1-50)complex. Protein Data Bank. https://www.rcsb.org/structure/7SNV. Deposited 26 October 2021.

