# Supplementary material for: Identification of a carbonic anhydrase–Rubisco complex within the alpha-carboxysome
Source: Proc Natl Acad Sci U S A. 2023 Oct 20;120(43):e2308600120. doi: 10.1073/pnas.2308600120 (PMC10614612; doi:10.1073/pnas.2308600120)
Supplement: Supplementary file 1 — Appendix 01 (PDF) [file pnas.2308600120.sapp.pdf]

PNAS

Supporting Information for

Identification of a carbonic anhydrase-Rubisco complex within the alpha-carboxysome

Cecilia Blikstad[a,c,1], Eli J. Dugan[a], Thomas G. Laughlin[a], Julia B. Turnšek[a], Mira D. Liu[b], Sophie R. Shoemaker[a], Nikoleta Vogiatzi[c], Jonathan P. Remis[d] and David F. Savage[a,e,1]

Affiliations:
[a]Department of Molecular and Cell Biology, University of California, Berkeley, California 94720, United States
[b]Department of Chemistry, University of California, Berkeley, California 94720, United States
[c]Department of Chemistry - Ångström Laboratory, Uppsala University, 75120 Uppsala, Sweden
[d]California Institute for Quantitative Biosciences (QB3), University of California, Berkeley, California 94720, USA
[e]Howard Hughes Medical Institute, University of California, Berkeley, California 94720

[1]To whom correspondence may be addressed. Email: cecilia.blikstad@kemi.uu.se or savage@berkeley.edu.

This PDF file includes:

- SI Figure 1 to 13
- SI Table 3
- SI Methods

Other supporting materials for this manuscript include the following:

- Dataset S01: Sequences for MSAs
- Dataset S02: sfGFP encapsulation
- Dataset S03: BLI results
- Dataset S04: Plasmids and proteins

Table of contents

Supplementary Figures 3

SI Fig. 1: Phylogenetic tree of CsoSCA 3
SI Fig. 2: CsoSCA-Rubisco interaction controls 4
SI Fig. 3: Binding of the NTD_{1-50}CsoSCA peptide to immobilized Rubisco 6
SI Fig. 4: Single-particle Cryo-EM data collection and processing workflow 7
SI Fig. 5: Resolution estimates and visual quality of Rubisco-NTD_{1-50}CsoSCA complex cryo-EM reconstructions 8
SI Fig. 6: Comparison of the two reconstructed Rubisco-NTD_{1-50}CsoSCA complex states 9
SI Fig. 7: Comparison of cryo-EM and crystal structures of Hnea CbbL/S 10
SI Fig. 8: Secondary structure prediction of NTD_{1-50}CsoSCA peptide and Rubisco sequence comparison 11
SI Fig. 9: Putative ordered waters mediating interaction between NTD_{1-50}CsoSCA peptide and Rubisco 12
SI Fig. 10: BLI sensograms of NTD_{1-53}-sfGFP and Rubisco point mutants 13
SI Fig. 11: Condensate formation with CsoS2, Rubisco and CsoSCA 14
SI Fig. 12: Possible binding conformations of CsoSCA-Rubisco 15
SI Fig. 13: Conservation analysis of CsoSCA NTD Rubisco binding motif 16

Supplementary Table 17

Table S1: Cryo-EM data collection, refinement and model statistics 17

SI Methods 19

Protein expression and purification full protocol 18
Growth phenotypes of *H. neapolitanus* csoSCA mutants 19

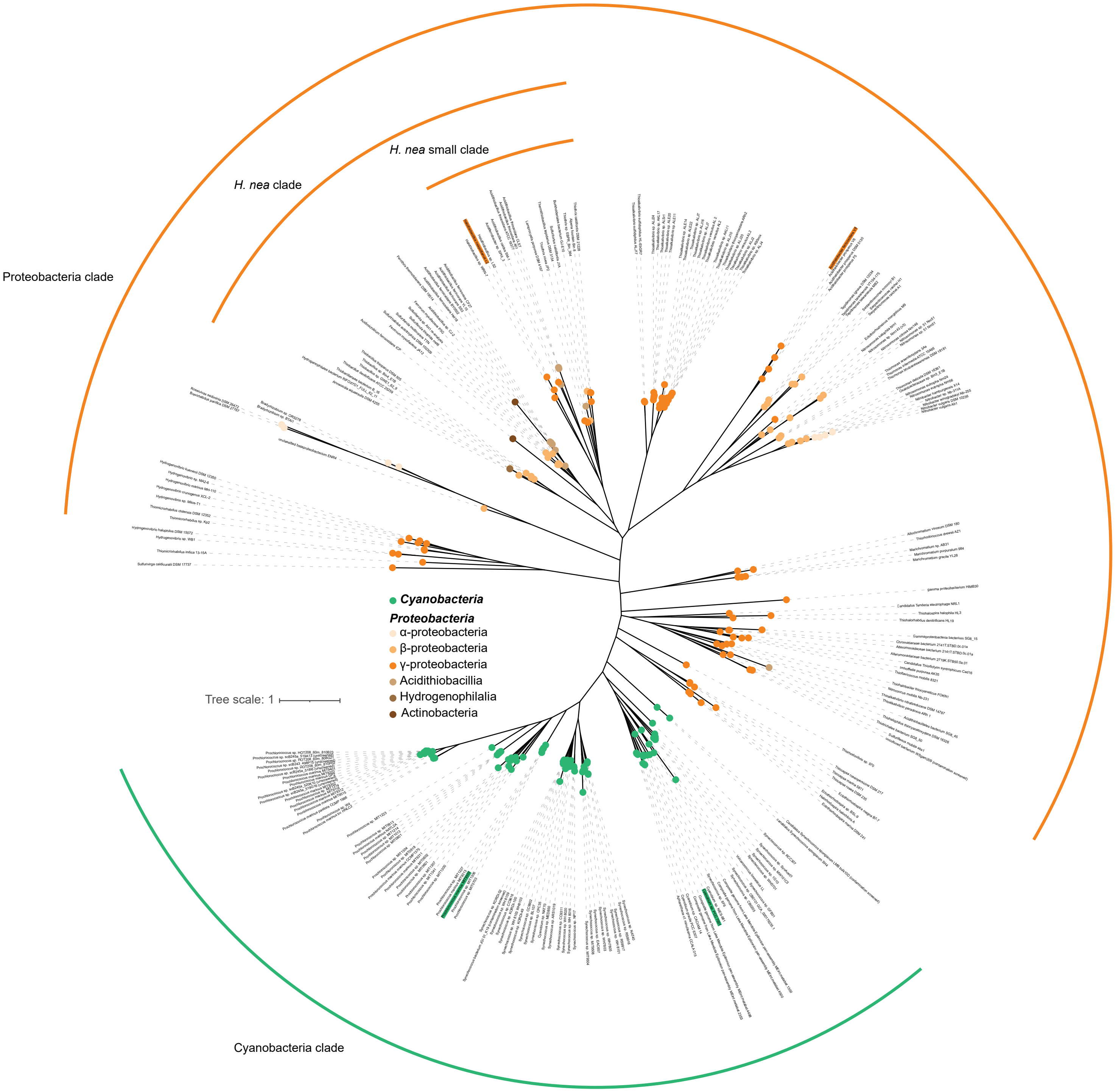

SI Figure 1: Phylogenetic tree of CsoSCA

Annotated maximum-likelihood phylogenetic tree of CsoSCA. Cyanobacterial homologs are colored in green and proteobacteria homologous in an orange/brown gradient. Scale bar, 0.1 substitutions per site.

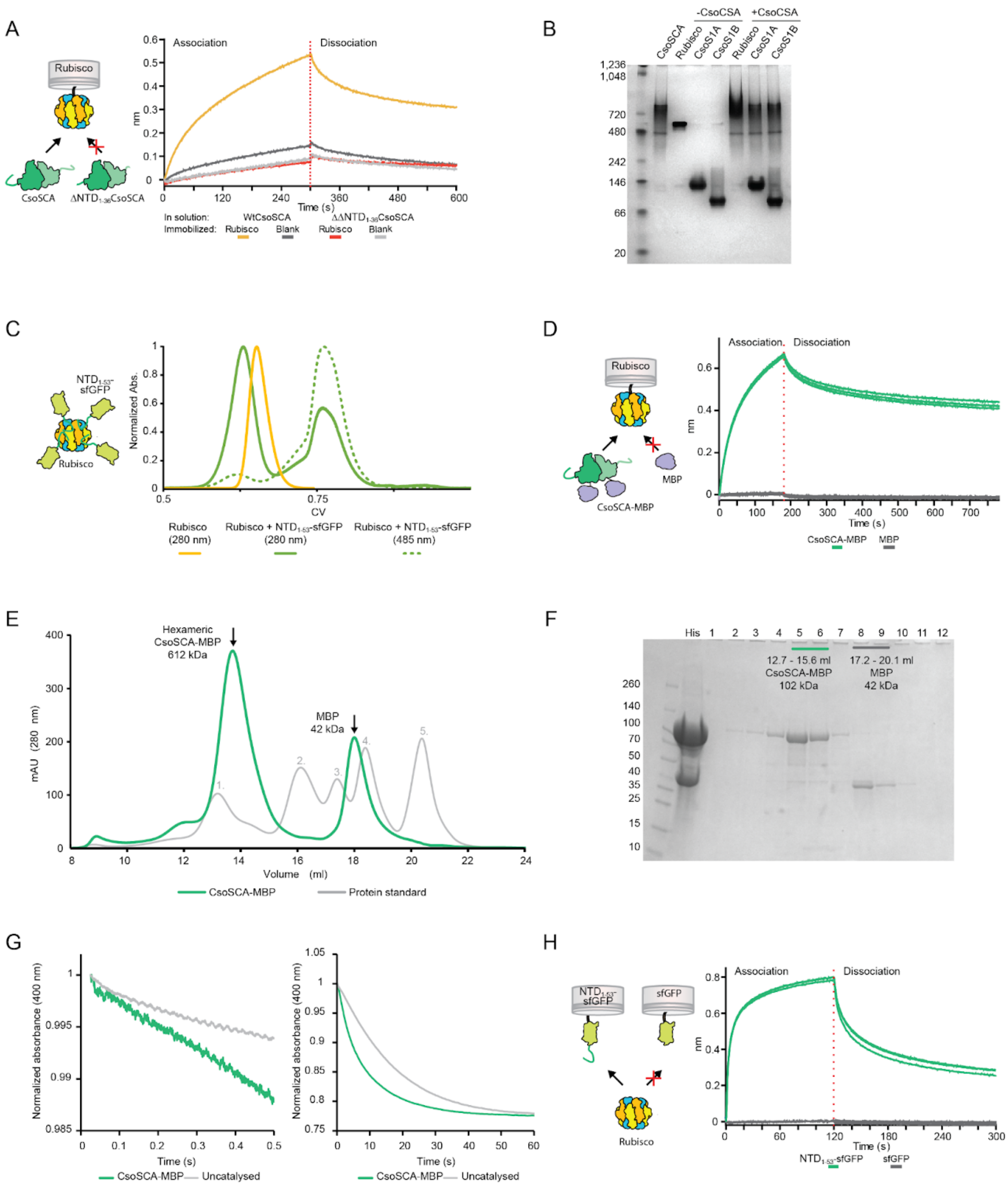

SI Figure 2: *CsoSCA-Rubisco interaction controls*

(A) BLI response showing that full length CsoSCA binds to Rubisco but a NTD truncated version ($\Delta NTD_{1\text{-}37}CsoSCA$) does not bind. (B) Native-PAGE demonstrating binding between Rubisco and CsoSCA-MBP, and lack of binding between the major shell proteins CsoS1A and CsoS1B. Experiments were repeated in triplicate. (C) Size exclusion chromatography experiment showing co-elution of Rubisco and $NTD_{1\text{-}53}$-sfGFP, demonstrating the interaction in a solution based assay. (D) BLI response showing that CsoSCA-MBP binds to Rubisco but not the negative control MBP. (E) Purification and determination of the oligomeric state of CsoSCA-MBP. Size exclusion-chromatogram of His-purified CsoSCA-MBP and Bio-Rad calibration standard. This is the second step of the purification and removes MBP-His contamination resulting in a pure CsoSCA-MBP complex. Expected molecular weight of the complex was

determined to ~600 kDa, indicating a trimer of dimer oligomeric state of CsoSCA-MBP. Molecular weight of standard was as follows: 1. Thyroglobin (670 kDa) 2. γ-globulin (158 kDa) 3. Ovalbumin (44 kDa) 4. Myoglobin (17 kDa) and 5. Vitamin B12 (1.4 kDa). (F) SDS-PAGE gel on fractions from Size exclusion-chromatogram of His-purified CsoSCA-MBP. Marked fractions correspond to pure CsoSCA-MBP (Green, #5-6) and MBP (Grey, #8-9) peaks on chromatogram in (E). (G) CO_2 hydration catalyzed by CsoSCA-MBP measured by the stopped-flow based carbonic anhydrase activity assay (the Khalifa/pH-indicator method), demonstrating that the CsoSCA-MBP fusion is catalytically active. Steady-state kinetics is measured over 0.5 s (left panel), and reaction equilibrium is measured over 60 s (right panel). (H) BLI response showing that Rubisco binds to NTD_{1-53}-sfGFP but not to the negative control sfGFP.

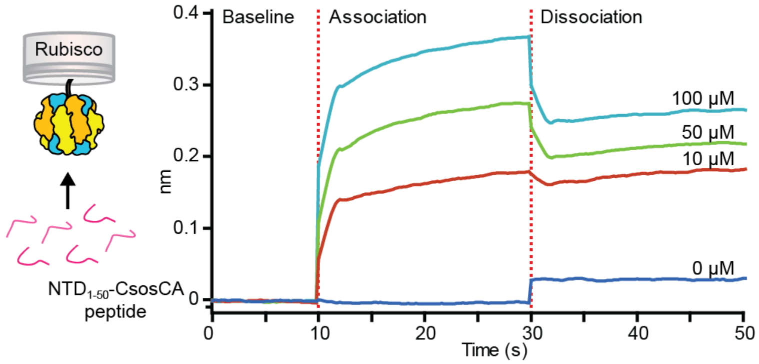

SI Figure 3: ***Binding of the $NTD_{1\text{-}50}$CsoSCA peptide to immobilized Rubisco***
BLI response showing binding of CsoSCA's $NTD_{1\text{-}50}$ peptide to Rubisco. Binding was measured with three peptide concentrations (10, 50 and 100 µM peptide).

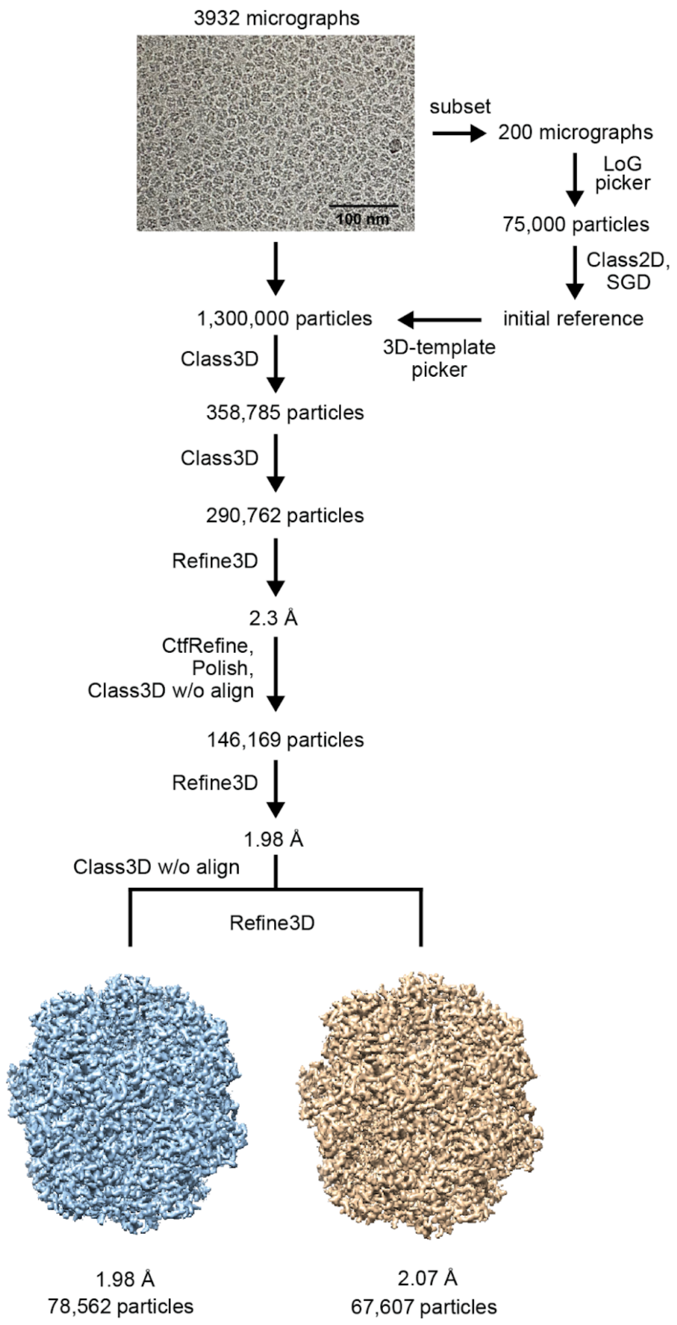

SI Figure 4: ***Single-particle Cryo-EM data collection and processing workflow***
Representative micrograph of the Rubisco-$NTD_{1\text{-}50}$CsoSCA complex and schematic of pre/processing, classification and refinement procedures used to generate the obtained in this study *see Methods for details).

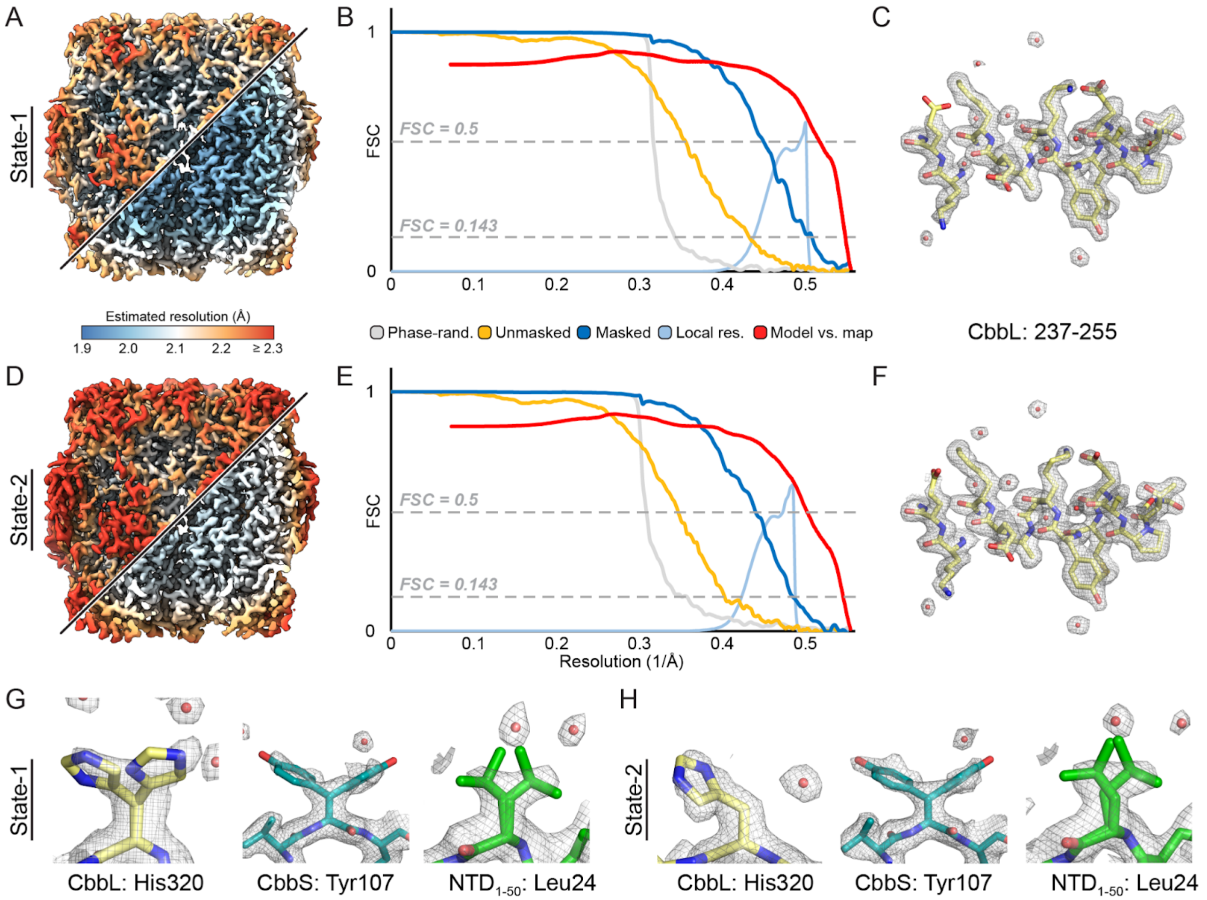

SI Figure 5: ***Resolution estimates and visual quality of Rubisco-NTD$_{1-50}$CsoSCA complex cryo-EM reconstructions***

(A) Local resolution estimate of State-1 map. The map is shown in unsharpened and locally filtered by estimated resolution. (B) Half-map and map-model FSC curves for State-1 map. (C) Exemplar State-1 density of an internal helix of CbbL from the density-modified and sharpened map contoured at 2σ. (D-F) Same as A-C for the State-2 cryo-EM reconstruction. (G,H) Model and density for residues with resolved alternate rotamer confirmations in either State-1 or State-2 reconstructions. Density shown is from the respective density-modified and sharpened map contoured at 2σ.

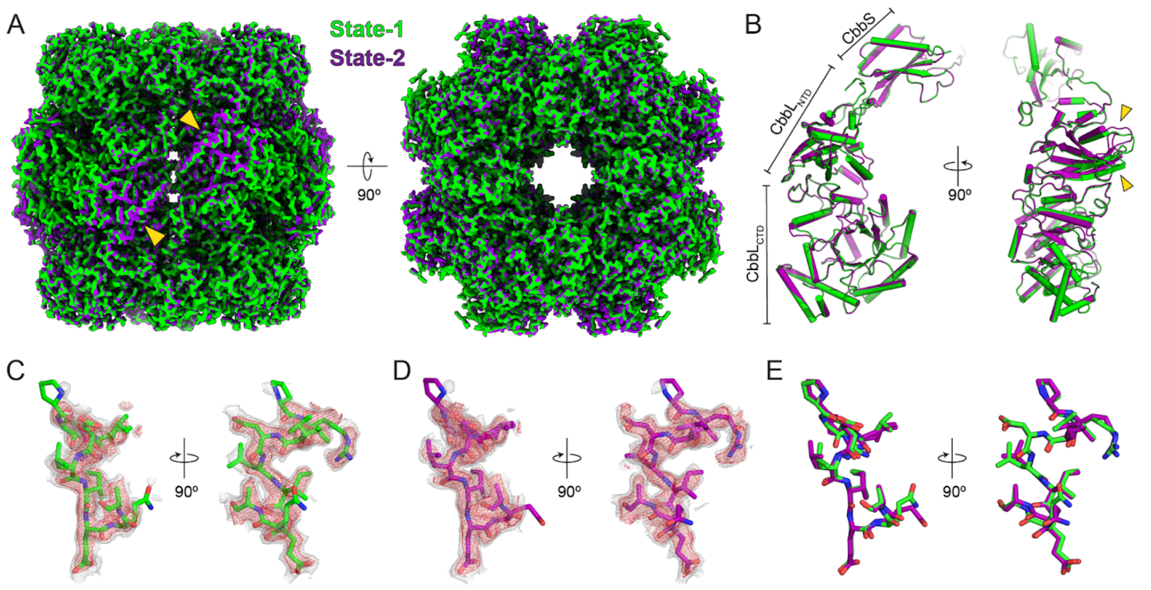

SI Figure 6: ***Comparison of the two reconstructed Rubisco-NTD$_{1\text{-}50}$CsoSCA complex states***

(A) Overlay of the two cryo-EM maps with State-1 in green and State-2 in purple. Yellow arrows point to regions of noticeable difference. (B) Asymmetric units of coordinate models for the two states, colored the same as in A. Subunits and domains are labeled and yellow arrows point to differences in loop confirmation in the CbbL$_{NTD}$. (C) State-1 NTD$_{1\text{-}5}$ peptide coordinate model and density contoured at 1.5σ (grey) and 2σ (red) from the density-modified, sharpened map. (D) Same as in C for State-2. (E) Overlay of NTD$_{1\text{-}50}$ peptide coordinate models of the two states.

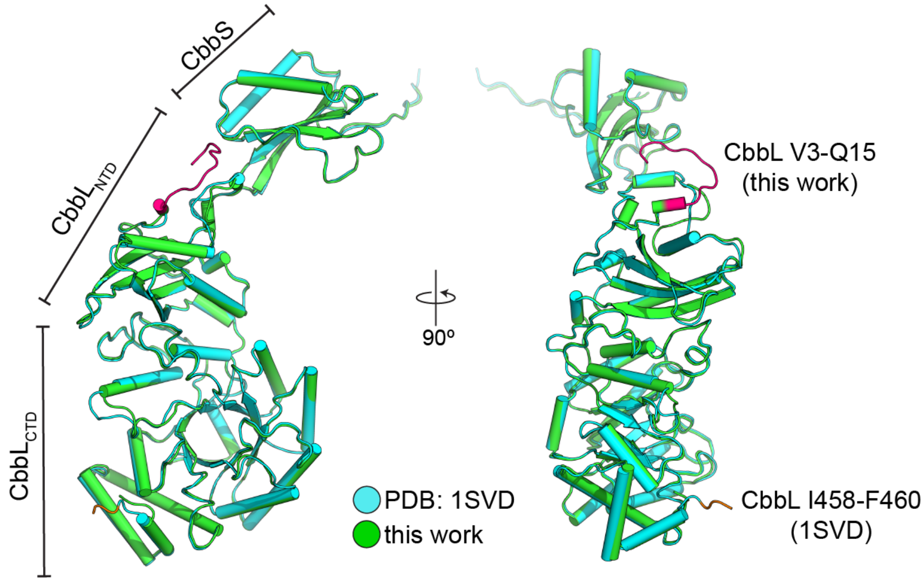

SI Figure 7: ***Comparison of cryo-EM and crystal structures of Hnea CbbL/S***

Overlay of CbbL/S from State-1 (this work) in green and the previous X-ray crystal structure (PDB:1SVD) in cyan. Distinct subunits and domains are labeled. Differences in the extent of resolved termini of CbbL between these structures are highlighted. Magenta coloring indicates additional N-terminal CbbL residues resolved in this work. Orange coloring indicates C-terminal CbbL residues resolved in the crystal structure.

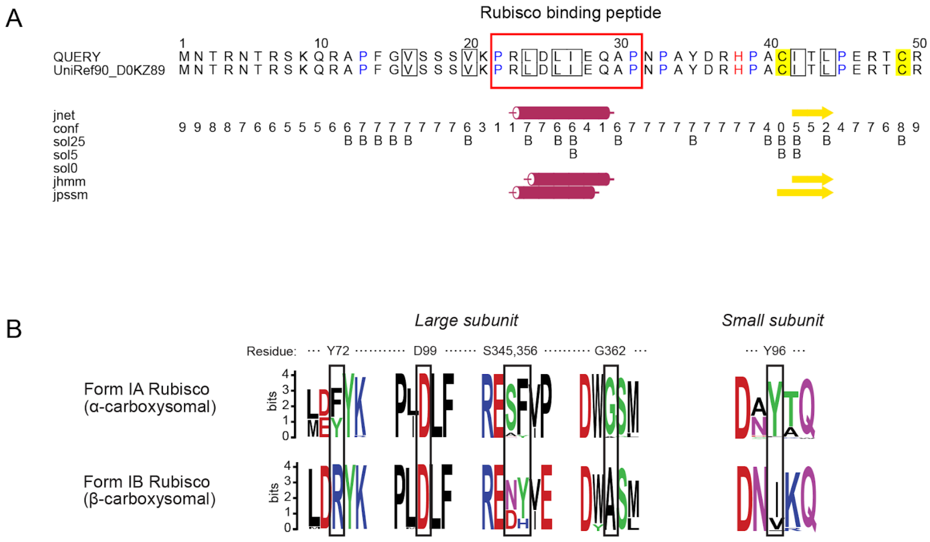

SI Figure 8: ***Secondary structure prediction of NTD$_{1\text{-}50}$CsoSCA peptide and Rubisco sequence comparison***

(A) JPred4 protein secondary structure prediction server was used to predict the secondary structure of *H. neapolitanus* CsoSCA's NTD$_{1\text{-}50}$ sequence. The PRLDLIEQAP sequence is predicted to form an alpha-helical structure (red cylinder). This stretch of sequence corresponds to the extra density observed in the cryo-EM structure of Rubisco in complex with the NTD$_{1\text{-}50}$ peptide (red box). (B) Rubisco sequence comparison at the CsoSCA-peptide interaction site. Multiple sequence alignment of α-carboxysomal Form IA Rubiscos and β-carboxysomal Form IB Rubiscos visualized using Weblogo (Dataset S1). Residues are numbered according to the Rubisco *H. neapolitanus* sequence. Residues shown in our Rubisco *H. neapolitanus* structure to interact with the CsoSCA peptide are marked with a black box. CsoSCA interacting residues have a high conservation score but are, in general, not conserved between α-carboxysomal and β-carboxysomal Rubiscos.

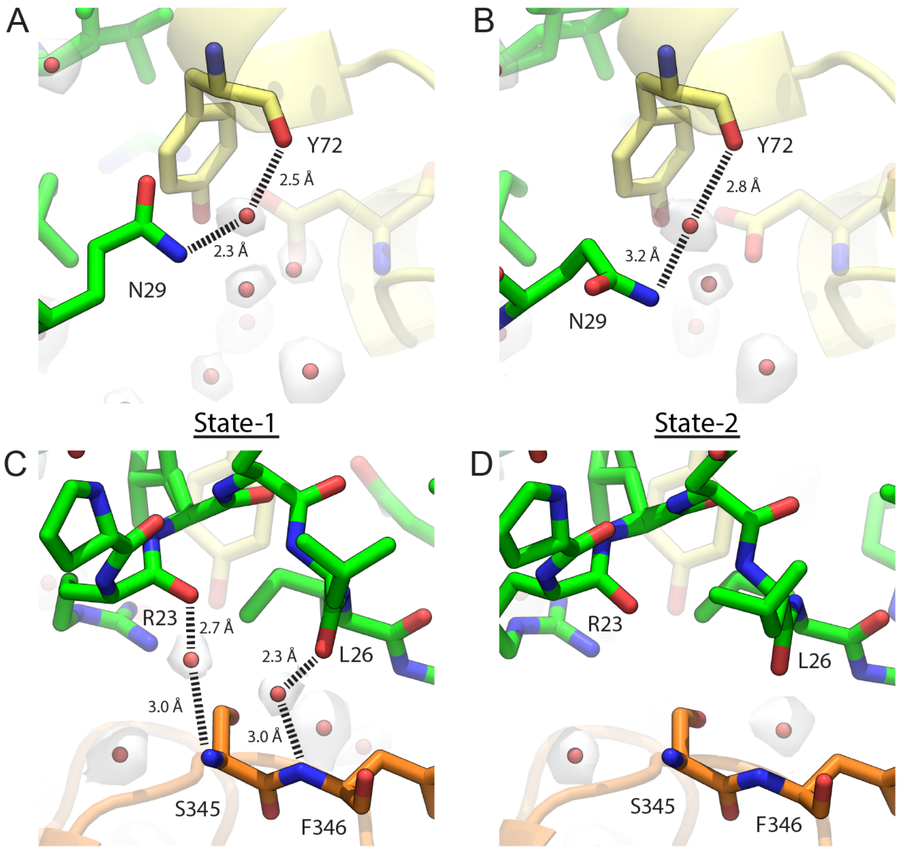

SI Figure 9: ***Putative ordered waters mediating interaction between $NTD_{1\text{-}50}$CsoSCA peptide and Rubisco***
(A,B) Water-mediated interaction between CsoSCA's $NTD_{1\text{-}50}$ peptide and $CbbL_A$ resolved in both Rubisco-$NTD_{1\text{-}50}$. (C,D) Water-mediated interactions between $NTD_{1\text{-}50}$ peptide and $CbbL_B$ resolved in the State-1 (C, 1.98 Å) map but not in State-2 (D, 2.07 Å) map. Putative ordered water densities are shown as transparent white surfaces contoured at 1.5σ from respective density-modified and sharpened maps.

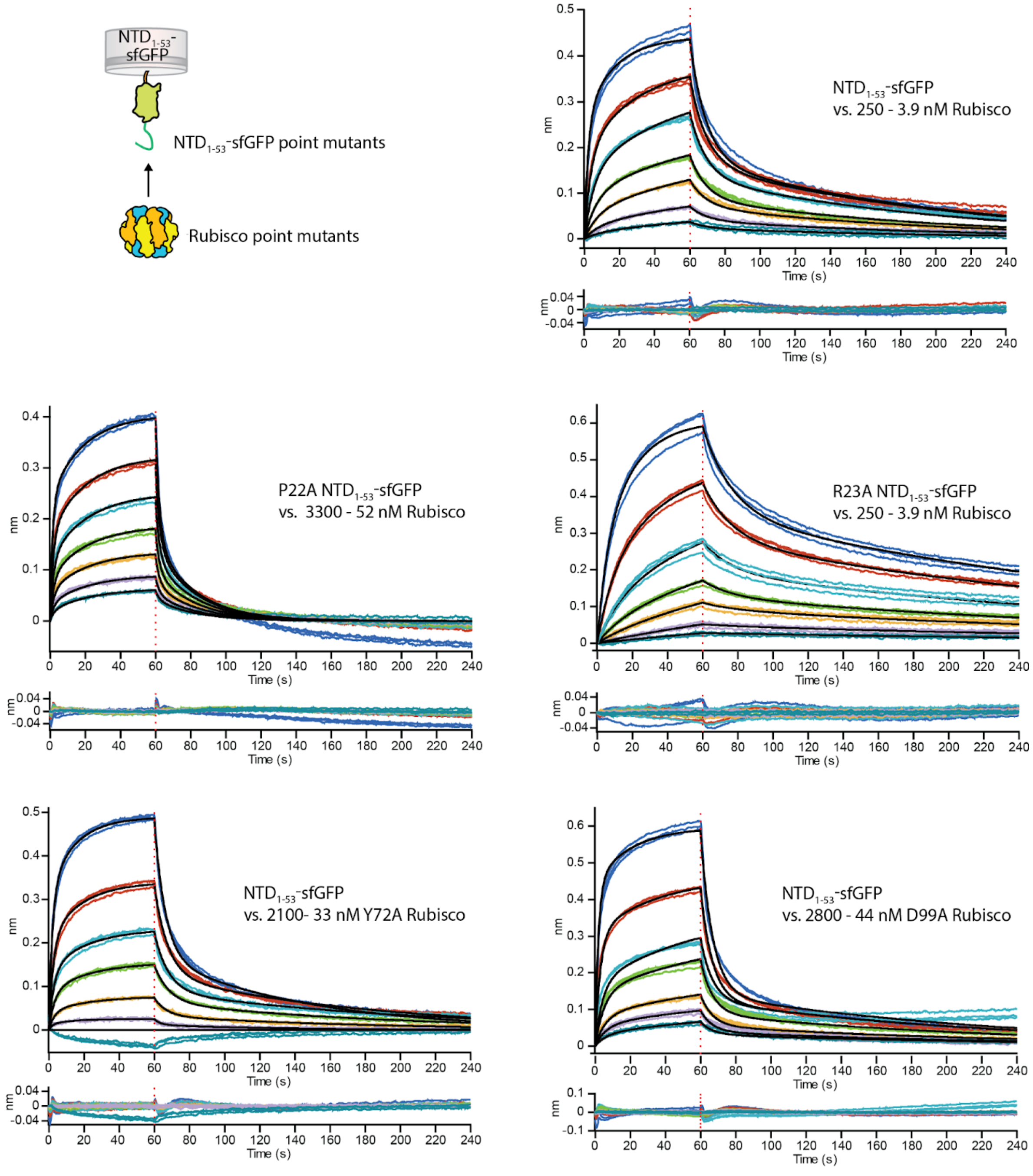

SI Figure 10: ***BLI sensograms of NTD$_{1\text{-}53}$-sfGFP and Rubisco point mutants***

BLI response from binding affinity measurements of Rubisco against immobilized NTD$_{1\text{-}53}$-sfGFP using point mutants of Rubisco or of NTD$_{1\text{-}53}$-sfGFP. Mutant and concentration range of Rubisco are indicated in the figure. K_D, k_{on} and k_{off} are listed in Table 1 and Dataset S3.

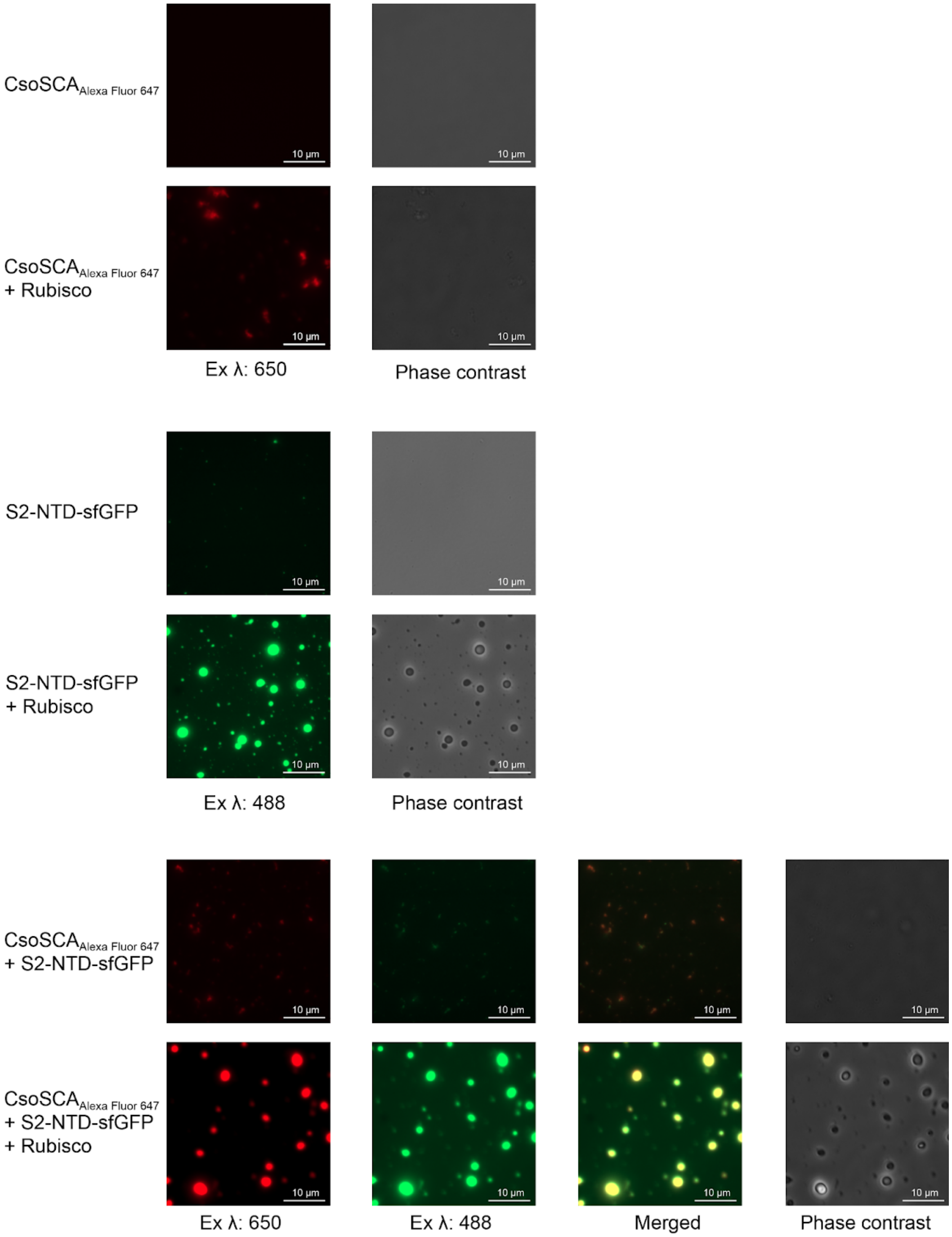

SI Figure 11: ***Condensate formation with CsoS2, Rubisco and CsoSCA***
Alexa Fluor 647, sfGFP and merged fluorescence as well as phase contrast images of protein droplets formed from a solution of Rubisco, CsoS2-NTD-sfGFP and Alexa Fluor 647 labeled CsoSCA-MBP (CsoSCA$_{Alexa\ Fluor\ 647}$) and of negative controls. Micrographs show that CsoSCA recruits into Rubisco-CsoS2-NTD protein condensates.

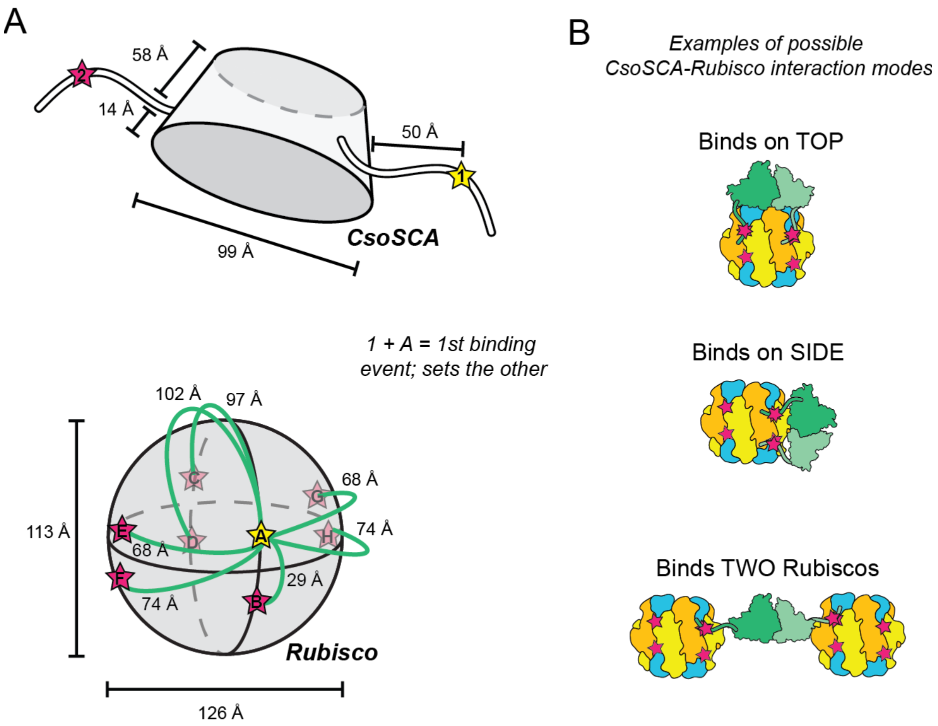

SI Figure 12: ***Possible binding conformations of CsoSCA-Rubisco***

(A) Diagram showing the approximate distances between binding motif on CsoSCA (top) and binding sites on Rubisco (bottom). Binding motifs/sites are marked with a star. (B) Cartoon representation of three examples of possible CsoSCA-Rubisco interaction modes.

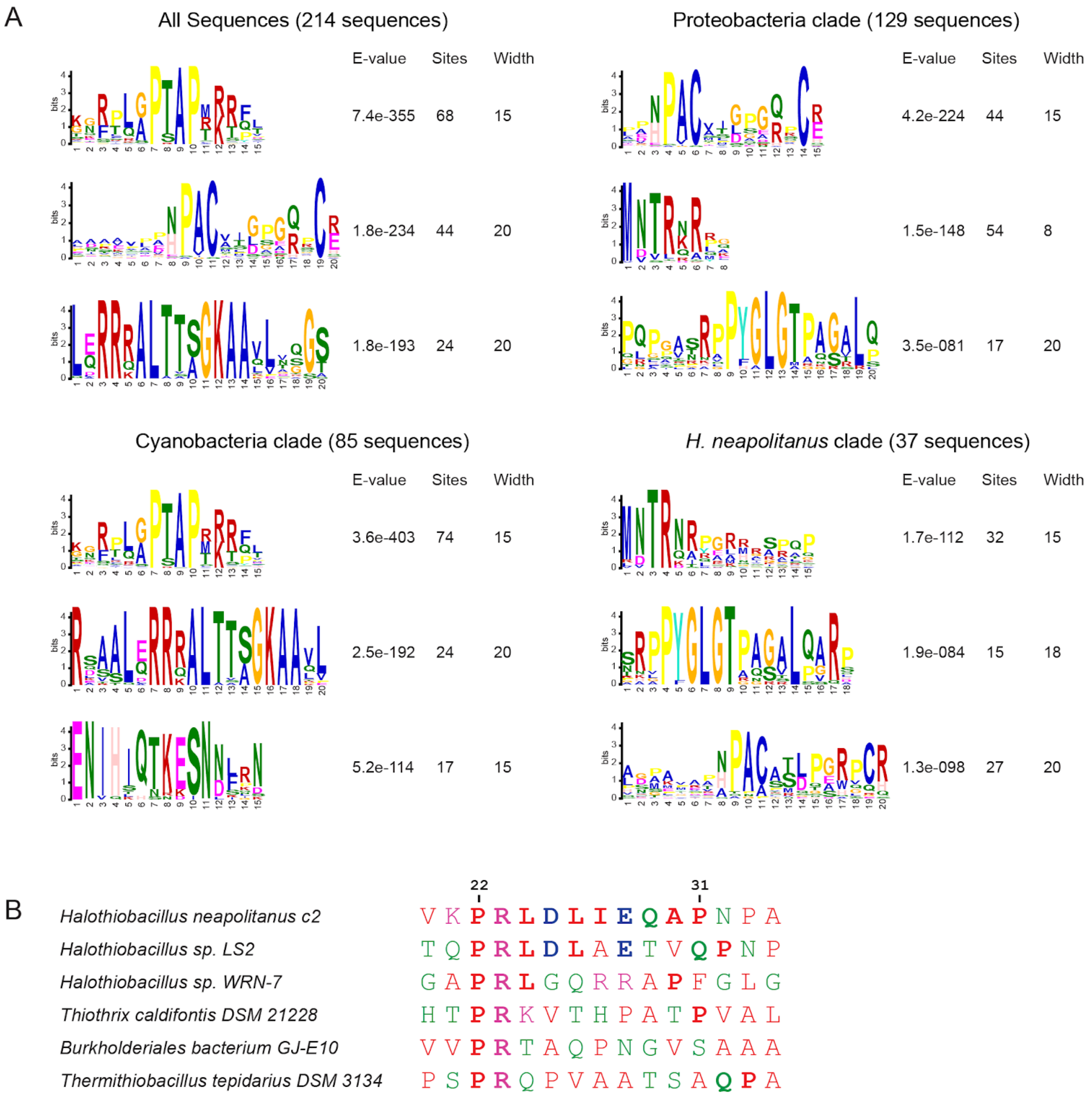

SI Figure 13: ***Conservation analysis of CsoSCA NTD Rubisco binding motif***

(A) Motif discovery of CsoSCAs NTD sequence using MEME analysis (Multiple Em for Motif Elicitation). Analysis was performed on all CsoSCA sequences, as well as sequences specific to the proteobacteria and cyanobacteria (Dataset S1). For a more stringent analysis, the *H. neapolitanus* clade was analysed separately as well. 44/129 of the collected proteobacteria sequences contain a PACxxxxxxxC motif. *H. neapolitanus* CsoSCA has this motif, however, it does not appear to be essential for binding to Rubisco. The N-terminus MNTRxR is somewhat conserved in proteobacteria (55/129 sequences). In cyanobacterial CsoSCAs a conserved PTAPxRR motif (75/85 sequences) is identified. 24/85 cyanobacteria sequences contain what appears to be the conserved CsoS2 Rubisco binding motif, RxxxxxRRRxxxxxGK. These sequences all belong to the *Procloroccocos* genus. The experimentally identified *H. neapolitanus* CsoSCA Rubisco binding motif, PRLDLIEQA, does not appear to be conserved among homologos. (B) Alignment of a selection of sequences from *H. neapolitanus* CsoSCAs closest homologs. Bold letters indicate residues conserved with the *H. neapolitanus* motif. Its closest homologue *Halothiobacillus sp. LS2* contains the same motif. Some of the others have the PR residues, demonstrated to be essential for binding, however the rest of the motif is poorly conserved.

Table S1: ***Cryo-EM data collection, refinement and model statistics***

	State 1 (ASU) (EMDB-25201) (PDB 7SMK)	*State 2 (ASU)* (EMDB-25228) (PDB 7SNV)
**Data collection and processing**		
Microscope	TFS Talos Arctica	
Detector	Gatan K3	
Voltage (kV)	200	
Nominal magnification	57,000	
Pixel size (Å)	0.69	
Electron exposure (e–/Å^2)	50	
Defocus range (μm)	-0.6 to -1.8	
Symmetry imposed	D4	
Initial particle images (no.)	~1.3 mil	
Final particle images (no.)	78,562	67,607
Map resolution (Å)	1.98	2.07
FSC threshold	0.143	
Map resolution range (Å)	1.9-2.7	2-2.9
**Coordinate model refinement**		
Initial model used (PDB code)	6UEW	
Model resolution (Å)	1.94	2.02
FSC threshold	0.5	
Map sharpening *B* factor (Å^2)	*phenix.autosharpen*	
Model composition		
Non-hydrogen atoms	4729	4685
Protein residues	571	571
Ligands (waters)	202	200
Atomic displacement parameters (Å^2)		
Protein	21.50	14.47
Ligand (waters)	22.68	13.10
R.m.s. deviations		
Bond lengths (Å)	0.008	0.007
Bond angles (°)	1.156	1.061
Validation		
MolProbity score	1.79	1.71
Clashscore	6.96	7.61
Poor rotamers (%)	2.15	0.88
Ramachandran plot (%)		
Favored	97.17	95.75
Allowed	2.65	4.07
Disallowed	0.18	0.18

SI Methods

Protein expression and purification full protocol

Specific details regarding *E. coli* strain, plasmid, and expression conditions for each protein in this study (CsoSCA variants, sfGFP-fusions, Rubisco, shell proteins and CsoS2) are provided in Dataset S4. Expression plasmids were transformed into either *E. coli* BL21-A1 or BW25113 cells (Dataset S4). For protein expression, cells harboring appropriate plasmids (Dataset S4) were grown at 37 °C in LB-medium supplemented with appropriate antibiotics. All Rubisco constructs were co-transformed with pGro7 for co-expression of GroEL/ES. At OD_{600} = 0.4 - 0.6 the temperature was decreased to 18 °C and expression induced by addition of 0.08% L-arabinose, 0.1 µM anhydrotetracycline (ATc) or 0.5 mM IPTG, see Dataset S4. Cells were grown overnight, harvested by centrifugation at 5,000 x *g* and frozen at -20 °C until use. Bacterial pellets were thawed and resuspended in appropriate binding buffer supplemented with 0.2 mM phenylmethanesulfonyl fluoride (PMSF), 0.1 mg/mL lysozyme and 0.1 µL/mL benzonase and lysed by three passes through a homogeniser (Avestin EmulsiFlex-C3) or by chemical lysis by diluting the resuspension 1:1 in B-PER II (Thermo Fisher) an incubating in RT for 30 min on a rocking table. Lysed cells were clarified by centrifugation at 27,000 x *g* for 45 min.

A summary of purifications of the proteins used in this study is described below. Specific details are provided in Dataset S4. All proteins were purified with either His-tag och Strep-tag affinity purifications. *His-tag purification (used for all CsoSCA-variants, sfGFP-fusions, shell proteins and CsoS2, see Dataset S4):* Clarified lysate was applied to a 5 mL HisTrap FF column (GE Healthcare) equilibrated with His-binding buffer (50 mM Tris, 300 mM NaCl, 20 mM imidazole, pH 8.0) using a syringe pump at a flow rate of 5 mL/min. Unspecific bound proteins were washed away with His-buffer containing 60 mM imidazole until A280 reached a stable baseline, and protein thereafter eluted with His-buffer containing 300 mM imidazole. *Strep-tag purification (used for Rubisco-variants, see Dataset S4):* Clarified lysate was applied to a 5 mL StrepTrap column (GE Healthcare) equilibrated with Strep-buffer (50 mM Tris, 300 mM NaCl, pH 8.0). Unspecific bound proteins were washed away with Strep-buffer until A280 reached a stable background, and protein thereafter eluted with Strep-buffer supplemented with 2.5 mM *d*-desthiobiotin (Sigma-Aldrich). All purified proteins were concentrated to appropriate concentration using 30 kDa Mw cut-off centrifugal filters (Amicon) and buffer exchanged into 50 mM Tris, 150 mM NaCl, pH 7.5 using 10DG Desalting Columns (Bio-Rad). *Untagged CsoSCA:* Untagged CsoSCA were purified as a His-SUMO fusion as outlined above. After elution from HisTrap column His-SUMO-CsoSCA was concentrated from 10 mL to 3 mL and buffer exchange into 50 mM Tris, 300 mM NaCl, 10% glycerol pH 8.0. The SUMO-tag was thereafter cleaved by addition of 1:200 molar ratio of Ulp-protease:His-SUMO-CsoSCA and 1 hour incubation. Cleaved protein mix was applied to a 1 mL HiTrap column to remove His-SUMO and residual uncleaved His-SUMO-CsoSCA. This resulted in a pure and untagged CsoSCA. Since full-length CsoSCA is prone to crash out in high protein concentrations care was taken to not exceed CsoSCA protein concentration above 1 mg/mL. *CsoSCA-MBP:* After His-tag purification protein was subsequently purified by size exclusion chromatography using a Superose 6

Increase 10/300 column (GE Healthcare). Protein purities were assessed by SDS-PAGE and were in general >95% pure. Protein concentrations were determined by A_{280} and the theoretically calculated extinction coefficient (ProtParam). All purifications were carried out at 4 °C. For storage, proteins were made to 10% (w/v) glycerol, flash-frozen in liquid nitrogen and stored in -80 °C. The oligomeric state of CsoSCA-MBP was determined from the Superose 6 Increase chromatogram and a Gel Filtration Standard (BioRad, #1511901). Molecular weight of standard was as follows: 1. Thyroglobin (670 kDa) 2. γ-globulin (158 kDa) 3. Ovalbumin (44 kDa) 3. Myoglobin (17 kDa) Vitamin B12 (1.4 kDa).

Growth phenotypes of *H. neapolitanus csoSCA* mutants

Generation of H. neapolitanus ΔcsoSCA and of WTcsoSCA and Δ$NTD_{1\text{-}49}$*csoSCA mutant complementations: csoSCA* was knocked out by transforming a plasmid containing a spectinomycin cassette with 1KB homology arms adjacent to *csoSCA* gene. Colonies that grew on spectinomycin were verified by colony PCR and Sanger Sequencing to verify a clean knockout. CsoSCA mutant complementations (*ΔcsoSCA+WTcsoSCA; ΔcsoSCA+*$NTD_{1\text{-}49}$*csoSCA)* were generated by transformation and concomitant genomic integration via homologous recombination into *H. neapolitanus* NS2 neutral site and verified by colony PCR and Sanger sequencing.

H. nea growth assays: Pre-cultures of WT *H. neapolitanus* and *H. neapolitanus ΔcsoSCA* were grown in DSMZ68 at 5% CO_2. *ΔcsoSCA* transformed with wild-type *csoSCA* or the N-terminal truncation *Δ*$NTD_{1\text{-}49}$*CsoSCA* were cultured in the same conditions with the addition of 1 µM IPTG to induce CA expression. All precultures were grown with the appropriate antibiotics. Upon reaching log phase, cultures were spun down for 15 minutes at 4,000 *x g* and pellets resuspended in 1 mL DSMZ68 without thiosulfate and pH indicator. Cultures were transferred into 1.7 mL eppendorf tubes and centrifuged at 4,000 x *g* for 8 minutes. This wash step was repeated twice before the cultures were diluted 5x and then normalized to a cell density of 0.1 OD600. Normalized cultures were serially diluted in 10x steps from 10^{-1} to 10^{-8} OD600. Resulting titers were spotted in 3 µL volumes onto plates with appropriate antibiotics in 5% CO_2 and ambient air; strains expressing complemented *WTcsoSCA* or *Δ*$NTD_{1\text{-}53}$*csoSCA* were plated on plates containing 1 µM IPTG. Strains were allowed to grow for 4 days. All strains were plated in biological and technical triplicate.
